# Repeated Reticulate Evolution in North American *Papilio machaon* Group Swallowtail Butterflies

**DOI:** 10.1371/journal.pone.0141882

**Published:** 2015-10-30

**Authors:** Julian R. Dupuis, Felix A. H. Sperling

**Affiliations:** Department of Biological Sciences, University of Alberta, Edmonton, Alberta, Canada; Virginia Tech, UNITED STATES

## Abstract

Hybridization between distinct populations or species is increasingly recognized as an important process for generating biodiversity. However, the interaction between hybridization and speciation is complex, and the diverse evolutionary outcomes of hybridization are difficult to differentiate. Here we characterize potential hybridization in a species group of swallowtail butterflies using microsatellites, DNA sequences, and morphology, and assess whether adaptive introgression or homoploid hybrid speciation was the primary process leading to each putative hybrid lineage. Four geographically separated hybrid populations were identified in the *Papilio machaon* species group. One distinct mitochondrial DNA clade from *P*. *machaon* was fixed in three hybrid taxa (*P*. *brevicauda*, *P*. *joanae*, and *P*. *m*. *kahli*), while one hybrid swarm (*P*. *zelicaon* x *machaon*) exhibited this hybrid mtDNA clade as well as widespread parental mtDNA haplotypes from both parental species. Microsatellite markers and morphology showed variable admixture and intermediacy, ranging from signatures of prolonged differential introgression from the paternal species (*P*. *polyxenes*/*P*. *zelicaon*) to current gene flow with both parental species. Divergences of the hybrid lineages dated to early- to mid-Pleistocene, suggesting that repeated glaciations and subsequent range shifts of parental species, particularly *P*. *machaon hudsonianus*, facilitated initial hybridization. Although each lineage is distinct, *P*. *joanae* is the only taxon with sufficient evidence (ecological separation from parental species) to define it as a homoploid hybrid species. The repetition of hybridization in this group provides a valuable foundation for future research on hybridization, and these results emphasize the potential for hybridization to drive speciation in diverse ways.

## Introduction

Hybridization between distinct populations or species has historically been considered trivial in the overall diversification of animal life (e.g. [1]) or as a countervailing force to speciation [2]. Modern molecular methods have turned this notion on its head, and it is becoming increasingly clear that hybridization is both more prevalent and evolutionarily more important than previously thought [3, 4, 5, 6, 2]. Hybridization can have a continuum of complex outcomes in speciation. It can inhibit speciation by homogenizing distinct populations through neutral diffusion [7], referred to as “breakdown” of reproductive isolation [8, 6]. When selection against hybrids limits neutral introgression between hybridizing populations, tension zones can form [9], with variable outcomes depending on the strength of selection. If selection is weak, tension zones can stabilize, thereby stalling speciation while maintaining genetic differentiation (e.g. [10]). If selection against hybrids is strong, reduced hybrid fitness can reinforce premating barriers (e.g. assortative mating), strengthening barriers to gene exchange and advancing speciation (“reinforcement” [11, 12]). Alternately, genetic differentiation can increase through adaptive introgression from one species to another [13], or unique admixture of new, hybrid species [14, 3, 15]. These phenomena are not mutually exclusive, and variation and combinations exist along the continuum. Ultimately, our interpretation of hybridization only captures “a single snapshot of a complex and continuously changing interaction” [2].

Homoploid hybrid speciation (hereafter referred to as hybrid speciation) and adaptive introgression are particularly important promoters of differentiation, as they can generate novel hybrid entities as well as the resources to fuel adaptive divergence of preexisting species [5, 2, 13]. Although both processes involve hybridization of distinct populations or species, hybrid speciation culminates with adaptive novel hybrid combinations forming a distinct and independent hybrid taxon [16, 3, 17]. In adaptive introgression, recombinant hybridization passes adaptive variation from one species to another, replacing less adaptive portions of the original genome, but maintaining the majority of that original genome [18, 19, 13]. Defining hybrid speciation thus necessitates identification of novel, hybrid traits or combinations that allow hybrids to be distinguished from parental taxa, regardless of the level of genetic admixture [2]. Identification of these traits can be difficult both theoretically and empirically, and is further complicated in systems exhibiting complex phylogenetic histories [20, 21], differential influences of parental taxa [15], and ancient or repeated hybridization [22]. However, complex systems that exhibit repeated hybridization at different temporal and spatial scales provide useful “natural laboratories” for understanding the ramifications of hybridization and downstream processes at an evolutionary scale [7, 23].

**Fig 1:**
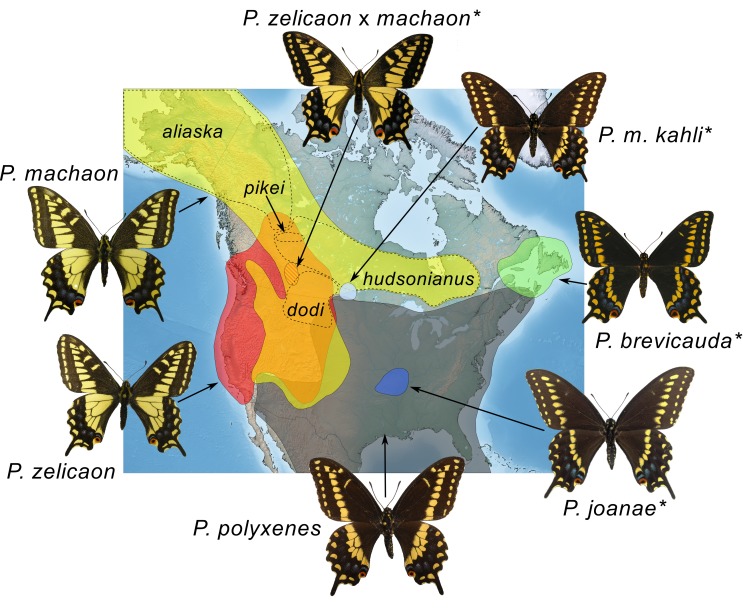
Generalized range map of current distributions of the *Papilio machaon* species complex in North America. Putative hybrid taxa are indicated with an asterisk. Dashed lines indicate approximate ranges of *P*. *machaon* subspecies pertinent to the current study. Map image: public domain from www.simplemappr.net, *Papilio joanae* holotype photograph: J. Tewell.

Swallowtail butterflies of the *Papilio machaon* species group (Lepidoptera: Papilionidae: the Old World swallowtails) provide a diverse model system in which to study hybridization. Species limits and systematic relationships have been notoriously difficult to resolve (e.g. [24, 25] in part due to a plethora of ecological races, color morphs, and incomplete reproductive barriers, as well as abundant natural hybridization [26, 27, 28]. Additionally, the genus as a whole has been influential in the development of many prominent theories in biology (speciation [29], the biological species concept [30], coevolution [31], mimicry [32], etc.), and has had a disproportionate influence on our understanding of the genetic and ecological dynamics of hybrid speciation [33, 34, 35]. In North America, six species are currently recognized within the *P*. *machaon* species complex: *Papilio machaon* (the only member to have a Holarctic distribution [26]), *P*. *polyxenes*, *P*. *zelicaon*, *P*. *brevicauda*, *P*. *joanae*, and *P*. *indra* [36]. *Papilio indra* is the only North American member of the species group to consistently have distinctive genitalia and adult wing pattern [28] and multiple genetic studies place it as the sister species to the rest of the clade [37, 38]. The five remaining North American species share many wing pattern characteristics, but can generally be separated into yellow- or black-morph species (Fig 1), although color polymorphism is widespread (e.g. [39, 40], also [26]). Larval hostplant use in the *P*. *machaon* group is confined to species of Asteraceae, Apiaceae (Umbelliferae), and Rutaceae, and while geographical specialization is the norm, uncommon species/hostplant pairings have been widely observed, suggesting that ecological differentiation is not strongly tied to larval hostplant constraints [26, 27].

The impetus for the research reported here was the finding that *P*. *brevicauda* and *P*. *joanae*, although morphologically quite similar to *P*. *polyxenes* (Fig 1), share mitochondrial DNA (mtDNA) restriction-site patterns with *P*. *machaon* [28]. Both *P*. *brevicauda* and *P*. *joanae* are geographically separated from *P*. *machaon*. *Papilio brevicauda* is restricted to the Maritime Provinces of Canada, often frequenting bluffs near the sea [41, 42]. *Papilio joanae*, on the other hand, is an endemic species of closed woodland habitats of the Ozark plateau in Missouri [43, 44], and because of its restricted range has been classified as “vulnerable” by conservation associations [45]. Both of these taxa have variably been considered species in good standing (*P*. *brevicauda* [46], *P*. *joanae* [36]) or subspecies/races of *P*. *polyxenes* due to their morphological resemblance (*P*. *brevicauda* [47], *P*. *joanae* [43]). More recently, however, they have been described as close relatives of *P*. *machaon* based on the mtDNA findings of [28] [48, 42, 36]. Mitochondrial DNA haplotypes identical to those in *P*. *brevicauda* and *P*. *joanae* were also found by [28] in putative hybrids in southwestern Alberta (*P*. *machaon* x *P*. *zelicaon* [26]) and southern Manitoba (*P*. *machaon kahli* [39], or *P*. *machaon* x *P*. *polyxenes* after [26]). Both of these populations represent morphological intermediates between the putative parental taxa, and while morphology and allozymes have been studied in the SW Alberta populations [26, 49], those in southern Manitoba have received less attention. Following [26] and [36], respectively, we refer to the populations in southwestern Alberta as *P*. *zelicaon* x *machaon*, and those in southern Manitoba as *P*. *machaon kahli*. “Parental taxa” refer to *P*. *machaon*, *P*. *polyxenes*, and *P*. *zelicaon*.

Despite the discordant mtDNA affinities of these taxa and their recognition as close relatives of *P*. *machaon*, no research has followed up on the hypothesis of ancient hybridization set out by [28]. Here we evaluate this putative hybridization across North America using mitochondrial and nuclear DNA sequences, microsatellite markers, molecular dating, morphological analysis of wing pattern, and assessment of ecological characteristics. Given the apparent evolutionary complexity of the group, we approach this evaluation using the preexisting taxonomic entities most prevalent in the literature (*sensu* [26, 36]) to guide our interpretation of these lineages, and ask to what extent each putative hybrid lineage (*P*. *brevicauda*, *P*. *joanae*, *P*. *m*. *kahli*, and *P*. *zelicaon* x *machaon*) exhibits characteristics of the putative parental species (*P*. *machaon*, *P*. *polyxenes*, and *P*. *zelicaon*). Using these data, we then evaluate whether significant support exists to classify each of these lineages as hybrid species, or if they appear to be the result of adaptive introgression. Following [2], we consider a hybrid species to be one that demonstrates novel characteristics or combinations that distinguish, and ideally, reproductively isolate the hybrid from *both* of its parents. Specifically, we use our genetic and morphological data to assess the genealogical discordance and distinctness of hybrid lineages, and focus on ecological separation to guide our inference of reproductive isolation (e.g. host plant, habitat, flight period, etc.). Genealogical and morphological distinctness, and reproductive isolation are considered to constitute significant evidence for the hypothesis of hybrid speciation.

## Materials and Methods

### Specimen selection and DNA extraction

Specimens were selected to represent the taxonomic breadth and diversity of the *P*. *machaon* species group in North America, as well as by the availability of DNA and wing vouchers. Species designations followed work on the same specimens or populations included in prior studies (e.g. [26, 28]). Morphology, geography, and in some cases larval host plant information were used to identify new specimens to species. We define species, in accordance with the genomic integrity species definition [50], as populations able to maintain their genomic integrity upon contact with each other, or allopatric populations with genetic divergence proportional to that found in closely related sister species. Slightly different subsets of specimens were used for each analysis due to DNA/wing quality limitations and voucher availability. Details are described below and species-specific sample sizes for each analysis are given in Table 1. All specimens used in this study, as well as associated locality and voucher deposition information, are given in Table A in S1 File (some geographic coordinates for private land and historic samples are not displayed). Collecting of recent material in provincial parks in Alberta and British Columbia was conducted under permit numbers 10–097 and 105180, respectively, and collecting on private land was done with the owner’s permission. All freshly collected material was collected with an aerial net (for adults), or hand collected as larvae and reared to the adult stage, then killed and stored at -70°C.

**Table 1:** Species and specimens examined in this study. Sample sizes for COI/COII sequence data (SEQ), microsatellites (MSAT), and morphological characters (MORPH). Number in parentheses in sequence data column corresponds to EF-1α.

Species	Subspecies	Seq	Msat	Morph
*P*. *machaon*	*aliaska*	13(1)	12	12
	*bairdii*	7(1)	4	7
	*dodi*	7	7	7
	*gorganus*	2(1)	2	2
	*hippocrates*	2(1)	2	3
	*hudsonianus*	7	6	6
	*oregonius*	1(1)	2	3
	*pikei*	8(1)	6	6
	*kahli*	3	4	3
*P*. *polyxenes*	*americus*	2(1)	3	4
	*asterius*	8(1)	11	9
	*coloro*	5(1)	6	6
	*stabilis*	1(1)	1	1
*P*. *zelicaon*		25(4)	24	19
*P*. *zelicaon* x *machaon*		23(1)	23	20
*P*. *brevicauda*	*brevicauda*	4	4	4
	*gaspeensis*	3(2)	2	3
*P*. *joanae*		6(2)	7	7
*P*. *hospiton*		1(1)	0	1
*P*. *indra*	*indra*	3(3)	3	3
	*kaibabensis*	1(1)	1	1
*P*. *xuthus*		1(1)	0	0
Total		*133(25)*	*130*	*127*

Sequence data generated by [51, 52, 53, 54] was retrieved from GenBank for a number of specimens within the *P*. *machaon* species group (Table 1), as well as *P*. *xuthus*, which served as an outgroup in the phylogenetic analyses [53]. Data retrieved from these sources varied from the full COI/COII genes (including the intervening *tRNA-leucine* gene; 2288 bp) to partial COI (394 bp), and the full EF-1α gene (1010 bp). Additional mitochondrial sequence data was generated for this study from pre-existing DNA extractions representing the major mtDNA restriction-site haplotypes found in [28]. More recently collected butterflies were also sequenced for COI, and in these cases, adult butterflies (field collected or reared from field-collected larvae) were killed and stored at -70°C. Additional EF-1α data was also generated for a subset of specimens from each species, although due to the limited and discordant phylogenetic information content of this gene, sequencing was not pursued for the remainder of the specimens (see Results). Microsatellite analysis was attempted on all specimens for which sequence data was available, except for a handful of specimens for which no DNA remained (including the only available specimen of *P*. *hospiton*). All recent DNA extractions (for both sequence data and microsatellites) were carried out using Qiagen DNeasy® Blood & Tissue extraction kits (QIAGEN, Mississauga, Ontario, CAN) using leg or thoracic tissue.

### Sequence data

Polymerase chain reactions (PCRs) were conducted in 50 μL reactions with a Biometra TGradient thermal cycler (Biometra, Goettingen, DE), including the following reagents: for COI/COII, 5 μL 10x PCR buffer (Promega, Madison, WI, USA), 3 μL of 25 mmoles/μL MgCl2 (Promega), 1 μL of 10 mmoles/μL dNTPs (Roche, Switzerland), 2 μL of each forward and reverse primer in 5 pmol/μL concentrations, 1 μL of 5 U/μL *Taq* polymerase (QIAGEN), 1 μL DNA, and 35.5 μL autoclaved Millipore water; for EF-1α, all reagent quantities were identical except for 2 μL of MgCl2 and 36.75 μL Millipore water. Reactions were conducted with a hot start (introducing *Taq* Polymerase after the initial 2 minute, 94°C denaturation period) followed by 35 cycles of 94°C for 30 seconds, 45°C for 30 seconds, and 72°C for 1 minute for COI/COII, and 35 cycles of 94°C for 30 seconds, 55°C for 1 minute, and 72°C for 1.5 minute for EF-1α. All reactions were finished with a 7-minute final extension at 72°C. Primers used in this study are given in Table C in S1 File. PCR purification was conducted with either a Qiagen QIAquick® PCR purification kit or a Qiagen QIAEX II® agarose gel extraction kit (QIAGEN). Sequencing reactions were carried out in both directions using a DYEnamic™ ET terminator cycle sequencing kit (Amersham Pharmacia Botech, Cleveland, Ohio, USA), and either filtered through Sephadex-packed columns or ethanol precipitated before being dried, resuspended, and fractionated on either an ABI PRISM® 377 or 3730 automated DNA sequencer (Applied Biosystems, Foster City, California, USA). Sequences were aligned using Mesquite v2.75 [55] and ClustalW v2.0.12 [56] using default settings. Alignment quality was checked by eye, but major adjustments were not necessary due to the absence of indels and introns. For the EF-1α sequences, double peaks consistently observed in the electropherograms were assumed to be the result of heterozygotes, and were coded using IUPAC ambiguity codes. The ends of sequences were trimmed to facilitate collapsing strictly redundant haplotypes in MacClade v4.08a [57]. Although in some cases this removed variable characters from the matrix for COI/COII, overall topological patterns were not affected.

### Phylogenetic analyses

Phylogenetic analyses were conducted using multiple optimality criteria to ensure that the choice of analytical method did not bias conclusions. Unweighted, unordered maximum parsimony (MP [58]) searches were conducted in PAUP* 4.0b10-x8 [59], with heuristic strategy of 1000 replicates of random sequence addition (holding 10 trees per replication), tree-bisection and reconnection branch swapping (TBR [60]) and no limit to the maximum number of trees retained per replication. Bootstrapping [61] with heuristic strategy of 100 replicates of random sequence addition (holding 100 trees per replication), TBR, and a maximum number of 100 trees (of minimum score 1) retained per replication, was conducted to test node support. To test for incongruence between data sets, Templeton tests [62] of data heterogeneity were conducted in PAUP* on a subset of taxa (specimens that had data for both COI/COII and EF1α) to identify if one data set could statistically reject the topology of the tree given by the other data set. In these tests, 50% majority rule consensus trees of the individual complete data sets (from MP searches) were constrained to the alternative data set and evaluated using the PSCORES command in PAUP*.

Maximum likelihood (ML) analyses were conducted in GARLI v0.951-GUI [63] applying models of evolution as predicted by the Akaike Information Criterion [64] in jModelTest 2.1.1 [65, 66]. The following models of evolution were used: extended COI/COII and complete EF-1α: Transition model (TIM) + I; complete COI/COII and extended EF-1α: TIM + Γ. All parameter values were specified in GARLI, and 100 bootstrap replicates were conducted.

Bayesian inference (BI) was conducted in MrBayes v3.2 [67]. Transition models of evolution are not applicable in MrBayes so were simplified, as in [68], as follows for these analyses: extended COI/COII: general time reversal (GTR [69]) + I + Γ; complete COI/COII and extended EF-1α: GTR + Γ; and complete EF-1α: Hasegawa, Kishino, and Yano model (HKY [70]) + I. Two million generations were run with trees being sampled from both runs every 100 generations, and default chain settings/temperatures. No priors were specified. The average standard deviation of split frequencies was observed during the run and the potential scale reduction factors were observed after the run to ensure that independent simulations were converging (values should approach zero and one, respectively). Burn-in trees were estimated visually for both runs in the log-likelihood overlay plot, and 25% of sampled trees were removed for burn-in. Posterior probability (clade credibility) values were calculated in MrBayes, and a 50% majority rule consensus tree was constructed in PAUP* after removing burn-in trees.

### Microsatellite markers

Ten of 17 microsatellite loci developed by [71] were reliably amplified in all species of interest (Table B in S1 File). Reverse primers for six of these loci were “PIGtailed” to decrease non-template nucleotide addition that hinders genotyping [72]. Microsatellite amplification was conducted using universal fluorescently labeled M13 forward primers [73], and sequence-specific primers mixed in a ratio of 4:1 reverse primer: M13 tailed forward primer. PCR reactions were conducted in 15 μL volumes containing 1.5 μL 10x microsatellite PCR buffer, 1.5 μL of 25 mM MgCl_2_, 0.3 μL dNTPs, 0.48 μL 4:1 sequence specific primer mix, 0.48 μL universal fluorescent-labeled M13 primer, 0.2 μL *Taq* DNA polymerase (Pickard Laboratory, University of Alberta), and 2.5 μL DNA, under the following cycling conditions: 10 min at 94°C, 38 cycles of [30 sec at 94°C, 45 sec at 56°C or 57°C (Table B in S1 File)], and 45 sec of 72°C, and followed by a final extension of 10 min at 72°C. Amplified fragments were run on an ABI Prism 3730 Analyzer (ABI), with a Genescan® LIZ-500 size standard, and genotyped using Genemapper® v4.0 (ABI). Descriptive statistics and measures of population differentiation (F_ST_) were calculated using GENODIVE [74].

### Individual-based clustering

Bayesian clustering of individual microsatellite data was conducted in STRUCTURE [75] using an admixture model and independent allele frequencies for all analyses. A burn-in period of 150,000 Markov chain Monte Carlo (MCMC) generations was followed by 500,000 generations for *k* = 1 through *k* = 10, with 10 iterations for each value of *k*. The most likely number of genetic clusters was calculated by evaluating the likelihood of the data (lnP(D∣K) [75]) and Δ*k* [76] with the program STRUCTURE HARVESTER [77]. CLUMPP v1.1.2 [78] was used to average replicate runs for each *k* value. STRUCTURE was also run using putative parental taxa as training sets, species determinations as “population” priors, and on a dataset including *P*. *indra*, the outgroup. Results using either training sets or population priors did not differ from analyses without these conditions, so the latter are presented here. Analyses including *P*. *indra* are provided in Fig. B in S1 File. Sub-structure was assessed in the overall analysis (by calculating ancestry for suboptimal Δ*k* values), and by breaking the dataset up according to the overall *k* = 2 results where individuals with ≥ 70% *machaon*-like ancestry were treated separately from remaining individuals. These methods produced similar assessments of substructure: The latter are focused on in the results section, as they provided clearer demarcation of substructure, and the former are provided in Fig. C in S1 File.

To investigate relatedness between clusters, we also conducted discriminant analysis of principal components (DAPC [79]), which submits genetic data to a principal component analysis (PCA) before conducting discriminant analysis (DA) on those principal components. This multivariate discriminant method does not attempt to minimize Hardy-Weinberg and gametic equilibrium (as does STRUCTURE [75]), and is therefore potentially more suited to this style of phylogenetically-oriented sampling. In maximizing between- and minimizing within-group variability [79], DAPC has also been shown to be more powerful and accurate with hierarchical relationships [80], which might be predicted in situations of hybridization and differential introgression. We implemented DAPC in R v3.0.1 [81] using *adegenet* v1.3.1 [82]. To provide comparison with STRUCTURE, the *find*.*clusters* function was used with default parameters, retaining all principal components (PCs), to find the ideal *k* value.

To visualize relationships between clusters using DAPC, the *optim*.*a*.*score* function was used to determine the optimal number of PCs to retain in the DA. In this function, 25 full simulations (parameter *smart* = FALSE) of a preliminary DAPC run (retaining the number of PCs corresponding to one-third the sample size of the run) are reiterated by the *optim*.*a*.*score* function to determine the ideal number of PCs to retain. This optimal number of PCs was then used in the final DAPC. All discriminant functions were retained for all DAPCs. The *xvalDapc* function was also used as an alternative to *optim*.*a*.*score*, and presented consistently similar, though somewhat larger (approximately 10–15 additional PCs), values of the optimal number of PCs. Due to uneven sample sizes between clusters, and potential biases in the *xvalDapc* function in these cases, *optim*.*a*.*score*’s determination of the ideal number of PCs was used (though retaining *xvalDapc*’s ideal number of PCs did not change the overall clustering pattern). Additionally, *optim*.*a*.*score*’s smaller optimal number of PCs graphically clustered the groups with smaller samples sizes closer to the main groupings, allowing better determination of relationships. DAPC was also conducted on a dataset including *P*. *indra*, the outgroup, and those results are provided in Fig. D in S1 File.

### Morphometrics

Six wing morphometric characters were used as in [26] (pg 208–209): A) extent of yellow scaling in cell Cu2, in anal margin of dorsal hindwing, B) shape of pupil in anal eyespot of dorsal hindwing, C) extent of black scales between blue and red portions of anal eyespot of dorsal hindwing, E) extent of yellow scales in basal half of disc of ventral forewing, F) extent of yellow scales of postmedian yellow band in apical cell of ventral forewing, and G) number of cells with orange patch in postmedian area of ventral hindwing, plus one. Right wings were used, unless characters were only visible on left wings. The quality and preserved tissues of 20–30 year old voucher specimens limited the amount of useable characters to those on the wing, and wing length characters were ignored due to large amounts of missing data associated with worn/tattered specimens; some specimens for which DNA-based data were available were too damaged to be scored (Table 1). Multiple correspondence analysis (MCA), an alternative to PCA for categorical variables, was conducted in R v3.0.1 [81] with the package FactoMineR [83].

### Molecular dating

Molecular dating of the complete COI/COII data was implemented in BEAST v1.6.2 [84], using BEAUti to generate the associated *xml* input file. Due to the paucity of fossil papilionids (see [38]), secondary calibrations from previous molecular dating studies in the Papilionidae were used to calibrate the tree. Five calibration points (shown in Fig. A in S1 File), including the root calibration, were used from the soft-bound age estimates calculated by [38] (nodes 167, 168, 169, 171, and 172). These were applied using the *tmrca* prior with a uniform distribution. Additionally the *Site Model* prior was set to match that used for BI (GTR + Γ), and the *Clock Model* prior was estimated with a relaxed, uncorrelated lognormal clock [85]. Both the Yule [86] and Birth-Death [87] process speciation *Tree Priors* were used, but did not change the results appreciably; results from the Birth-Death process prior will be reported. Five independent runs of 100 × 10^6^ generations, being sampled every 1 × 10^3^ generations, were run and combined using LOGCOMBINER. To ensure proper parameter estimates, TRACER was used to check effective sample sizes. A burn-in of 25% of the trees from each run was removed before all combined trees were summarized in TREEANNOTATOR.

## Results

### DNA sequence properties

Sequence data was collected from 133 individuals representing 22 taxa (Table 1, GenBank accession numbers: Table A in S1 File). DNA alignments for the gene regions *cytochrome oxidase I/II* (COI/COII) and *elongation factor-1α* (EF-1α) were 2288 bp and 1010 bp respectively (Table 2), although most sequences were shorter than this total. To test whether missing data affected overall topology, all phylogenetic analyses were conducted with the “extended” dataset (including missing data) and with a “complete” dataset that included only shorter sequence regions present in all specimens. The shorter alignments consisted of 306 and 418 bp, for COI/COII and EF-1α respectively.

**Table 2:** Summary results from maximum parsimony and maximum likelihood analyses. Extended and complete data sets are included.

Gene	Data set	Characters		MP				ML Score
		Inf.*	Total	# Trees	Score	CI	RI	
COI/COII	Extended	164	2288	15186400	463	0.793/0.668	0.895	-5369.1201
	Complete	24	306	28	71	0.817/0.675	0.882	-753.4862
EF-1α	Extended	16	1010	22976344	86	0.919/0.720	0.877	-1927.2094
	Complete	9	418	859	42	0.952/0.833	0.957	-831.0601

Abbreviations: CI: consistency index, RI: retention index, Inf. *: parsimony informative characters, MP: maximum parsimony, ML: maximum likelihood. For CI values, the first reported number includes uninformative characters, and the second excludes them.

The COI/COII data included 133 specimens that were consolidated to 54 unique haplotypes in the complete dataset, and the EF-1α data included 27 specimens that contained 25 unique genotypes (Table 1, Table A in S1 File). Tests of topological incongruence concluded that the two data sets (COI/COII and EF-1α) were not homogenous. Constraining the abridged COI/COII data to the EF-1α topology supported incongruence between the datasets (*p* = 0.0002); reversing that constraint (i.e. constraining EF-1α data to COI/COII topology) did not (*p* = 0.0588), as could be expected since the EF-1α data contained relatively little variation, and hence phylogenetic information (Table 2), and had few resolved clades (see below).

### Phylogenetic relationships

**Fig 2:**
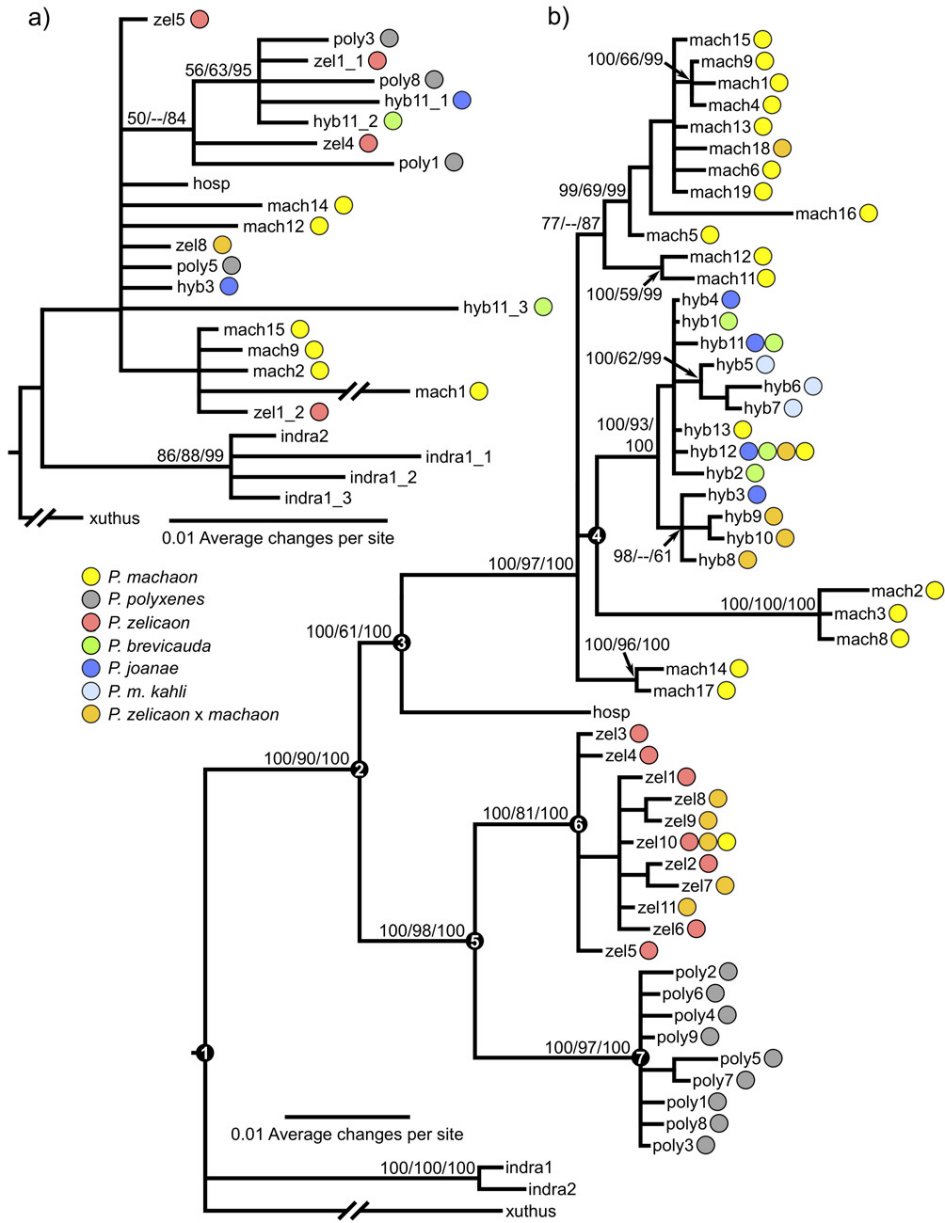
Fifty percent majority rule consensus trees constructed using Bayesian Inference. a) Extended EF-1α sequence data for 25 genotypes, and b) extended COI/COII sequence data for 54 haplotypes. Numbers above branches represent bootstrap support (if >50%) for maximum parsimony (first number), maximum likelihood (second number) and Bayesian posterior probability values (third number), for nodes that are consistently resolved among search criteria. Colors in circles correspond to the species possessing each haplotype. EF-1α genotype names reflect the corresponding COI/COII haplotype displayed by each specimen, with an added underscore and number indicating cases where specimens that shared COI/COII haplotypes had more than one different EF-1α genotype. Age estimates using COI/COII data for major nodes (numbered black circles) are provided in Table 4.

Maximum parsimony, ML, and BI analyses produced very similar, although not identical, topologies for each data set. Measures of branch support (MP and ML bootstrap and BI posterior probability) generally increased for phylogenies based on extended data for COI/COII, but decreased for EF-1α. Overall topology was not affected by the inclusion of regions of missing data. Bayesian 50% majority rule consensus trees are shown here for the extended datasets (Fig 2), and summary information for MP and ML are presented in Table 2.

COI/COII supported previously resolved relationships [26, 51, 53, 88, 38] between *P*. *machaon*, *P*. *hospiton*, *P*. *polyxenes*, and *P*. *zelicaon* (Fig 2B). The mitochondrial phylogeny failed to separate subspecies within *P*. *machaon* and *P*. *polyxenes* (Table A in S1 File) indicating high intraspecific variability or retained ancestral polymorphism within these species, which is discordant with patterns defined by morphology and geography. Complete fixation of one clade of *machaon*-like mtDNA was observed in *P*. *brevicauda*, *P*. *joanae*, and *P*. *m*. *kahli*, while *P*. *zelicaon* x *machaon* hybrid populations contained that clade as well as *machaon*- and *zelicaon*-like haplotypes more typical of the parental species. All putative hybrids except *P*. *m*. *kahli* showed within-group variability, and several haplotypes within the main hybrid clade were shared between species (e.g. hyb11 and hyb12). The main hybrid clade was monophyletic with regard to putative hybrid populations, except for the presence of two *P*. *machaon* haplotypes: hyb13 from a single *P*. *m*. *pikei* specimen collected in Alberta and hyb12 in several *P*. *m*. *hudsonianus* specimens collected in Manitoba and Québec (Fig 1). Interestingly, the sister lineage to the main hybrid clade is a highly supported, divergent (long branch lengths) clade belonging to two specimens of *P*. *m*. *aliaska* collected from northern British Columbia and Alaska (mach2 and mach3) and one specimen of *P*. *m*. *hudsonianus* collected from Manitoba (mach8). Additionally, two specimens identified as *P*. *m*. *aliaska* (based on morphology, flight period, and habitat) exhibited *zelicaon*-like mtDNA (zel10); upon further examination of these specimens, they showed several intermediate morphological characters between *P*. *m*. *aliaska* and *P*. *zelicaon*.

EF-1α sequences only supported the monophyly of *P*. *indra*, and gave no resolution for any other species within the group (Fig 2A). Due to the ambiguous phylogenetic information content of EF-1α and difficulty in consistently obtaining sequences from older DNA, comprehensive sequencing of all specimens was not pursued.

### Microsatellite data

**Fig 3:**
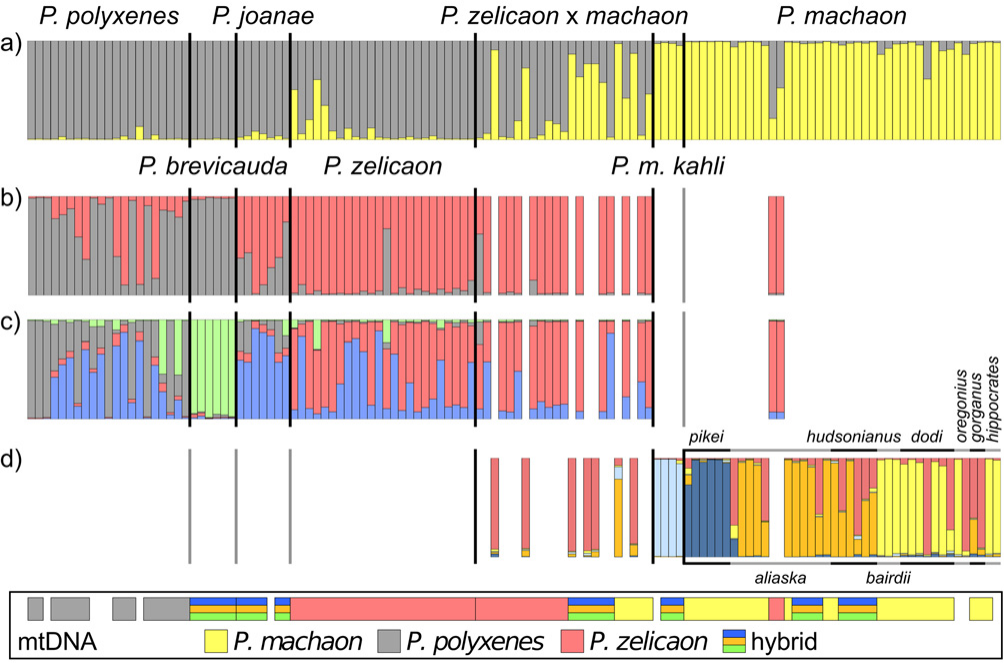
STRUCTURE results for microsatellite genotype data. a) *k* = 2 based on overall dataset; b) and c) *k* = 2 and *k* = 4, respectively, for the *polyxenes*/*zelicaon*-like cluster from the overall *k* = 2; and d) *k* = 5 for the *machaon*-like cluster from the overall *k* = 2 (individuals with ≥70% *P*. *machaon* ancestry). Inset indicates the major mtDNA clade (*P*. *machaon*, *P*. *polyxenes*, *P*. *zelicaon*, or the main hybrid clade within the *P*. *machaon* clade) for each individual (gaps indicate specimens genotyped for microsatellites that were not sequenced for COI/COII). Alternating black and grey bars above and below the *P*. *machaon* portion of d) indicate subspecies; from left to right: *P*. *m*. *pikei*, *aliaska*, *hudsonianus*, *bairdii*, *dodi*, *oregonius*, *gorganus*, and *hippocrates*.

Ten microsatellite loci were genotyped for 130 specimens representing 20 taxa (Table 1), and had a total of 225 alleles with a range of 11–32 alleles per locus (Table B in S1 File). Observed heterozygosity ranged from 0.25 in *P*. *indra* to 0.67 in *P*. *zelicaon* x *machaon* and pairwise F_ST_ ranged from 0.010 between *P*. *zelicaon* and *P*. *zelicaon* x *machaon* to 0.600 between *P*. *m*. *kahli* and *P*. *indra* (Table 3). STRUCTURE predicted an ideal *k* value of *k* = 2, with clusters roughly corresponding to 1) *P*. *machaon* including *P*. *m*. *kahli*, and 2) *P*. *polyxenes*, *P*. *brevicauda*, *P*. *joanae* and *P*. *zelicaon* (Fig 3A); individuals of *P*. *zelicaon* x *machaon* were split between the two main clusters or, along with some individuals of *P*. *zelicaon*, were intermediate. Sub-structuring was present for both overall clusters. Within the *polyxenes*/*zelicaon*-like cluster, *k* = 2 separated *P*. *polyxenes* and *P*. *brevicauda* from *P*. *zelicaon* and *P*. *joanae* (Fig 3B), and *k* = 4 identified *P*. *brevicauda* as having a unique signature (Fig 3C). Within the *machaon*-like cluster, *k* = 5 was best supported, which clearly distinguished *P*. *m*. *kahli* and *P*. *m*. *pikei*, but showed variable ancestry for the other subspecies of *P*. *machaon* and individuals of *P*. *zelicaon* x *machaon* from SW Alberta (Fig 3D). Two individuals of *P*. *m*. *aliaska* were *zelicaon*-like or *zelicaon* x *machaon*-like throughout the STRUCTURE results (Fig 3); these were the same two individuals that exhibited *zelicaon*-like mtDNA (Fig 2B).

**Table 3:** F_ST_ and heterozygosity values based on microsatellite data. F_ST_ comparisons based on microsatellite data between species in bottom triangle, heterozygosity values within each species on diagonal (observed/expected).

	*polyx*.	*brevi*.	*joanae*	*zelicaon*	*zel x mac*	*kahli*	*mach*.	*indra*
*P*. *polyxenes*	0.47/0.81	-	-	-	-	-	-	-
*P*. *brevicauda*	0.114	0.51/0.57	-	-	-	-	-	-
*P*. *joanae*	0.068	0.223	0.61/0.78	-	-	-	-	-
*P*. *zelicaon*	0.047	0.143	0.049	0.62/0.87	-	-	-	-
*P*. *zelicaon x machaon*	0.058	0.181	0.065	0.010	0.67/0.84	-	-	-
*P*. *m*. *kahli*	0.245	0.456	0.309	0.202	0.217	0.44/0.41	-	-
*P*. *machaon*	0.113	0.216	0.096	0.067	0.047	0.236	0.50/0.82	-
*P*. *indra*	0.281	0.481	0.375	0.239	0.291	0.600	0.288	0.25/0.43

**Fig 4:**
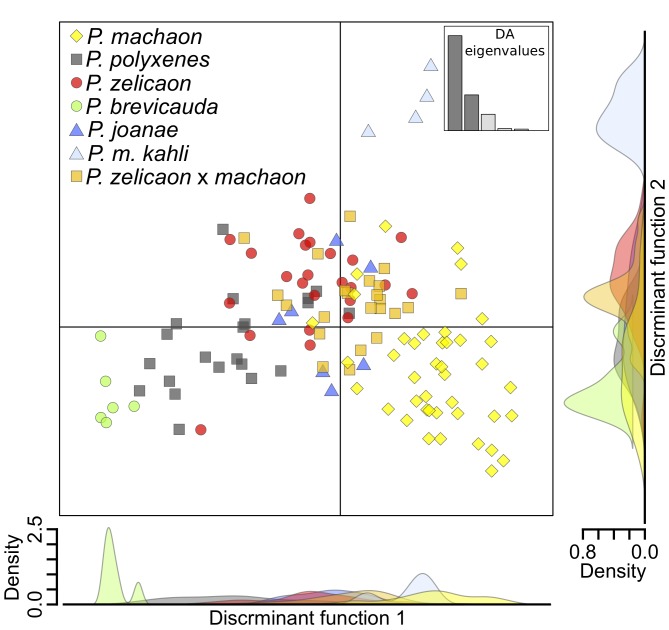
DAPC of putative hybrids and parental taxa generated from microsatellite data. Inset illustrates the relative contribution of individual discriminant functions (DFs) to overall variability, and the density plots of the two plotted DFs are shown outside of the main plot.

Discriminant analysis of principal components (DAPC) of microsatellite data optimally predicted *k* = 3 genetic clusters, corresponding to one group consisting of *P*. *machaon* and a few *P*. *zelicaon* x *machaon*, and two groups sharing all individuals of the other species and a small number of *P*. *machaon*. Graphically, there is no clear demarcation/separation between these groups; individuals instead fall along a gradient from *P*. *machaon* to *P*. *zelicaon* to *P*. *polyxenes* (Fig 4). *Papilio joanae* grouped between *P*. *polyxenes* and *P*. *zelicaon*, specimens of the *P*. *zelicaon* x *machaon* population were between their putative parental species, and *P*. *brevicauda* clustered closest to *P*. *polyxenes*, but with some separation from the main gradient. The contributions of individual discriminant functions are displayed as an inset in Fig 4, showing that the first function accounts for most of the variation. *Papilio machaon kahli* clusters among *P*. *machaon* on the first discriminant axis (the x-axis, discriminant function 1), but is separated from the main cluster along the second (the y-axis, discriminant function 2). This separation could be an artifact of small sample size capturing very little within-group variability, but generating additional “individuals” from random alleles observed in *P*. *m*. *kahli* maintains this overall pattern.

Considering the overall pattern of genetic clustering and observed intraspecific variation, STRUCTURE and DAPC produce similar results; namely, both analyses show optimal clustering of individuals that separates most *P*. *machaon* from *P*. *polyxenes*/*P*. *zelicaon*, although STRUCTURE illustrates this pattern more explicitly. It is clear that despite their mitochondrial relationships, *P*. *brevicauda* and *P*. *joanae* share little nuclear resemblance with *P*. *machaon*. *Papilio machaon kahli* appears *machaon*-like at a broad level, but along with *P*. *brevicauda*, is genetically distinct at a finer scale. Finally, *P*. *zelicaon* x *machaon* shows signatures of high and variable admixture in both STRUCTURE and DAPC, which is exemplified by the occurrence of *machaon*-like, *zelicaon*-like, and hybrid-like individuals present within each of the main mtDNA clades (Fig 3, inset).

### Morphometrics

**Fig 5:**
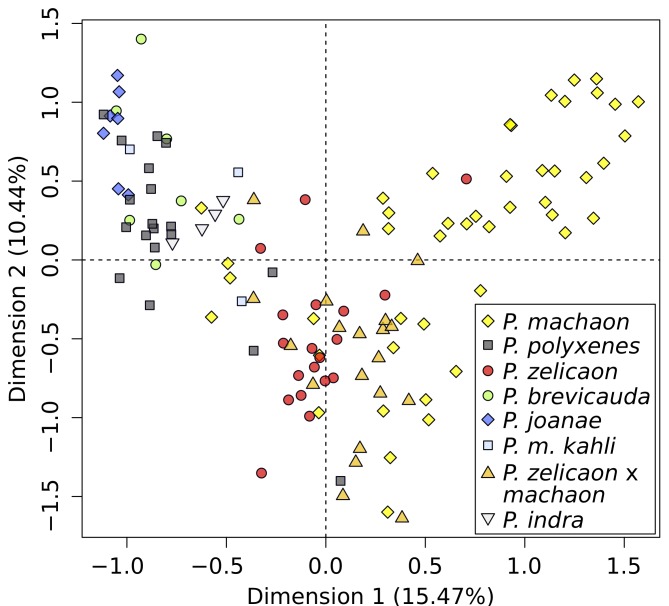
Multiple correspondence analysis of six morphological characters. Plotted using the *jitter* function to improve visualization of overlapping points (non-*jittered* results shown in Fig. E in S1 File).

Six wing morphometric characters from [26] were scored for 127 specimens representing 21 taxa (Table 1), and analyzed with multiple correspondence analysis (MCA) (Fig 5). Dimension one of the MCA created a gradient between the parental species, stretching from “yellow morph” species (*P*. *machaon*) to “black morph” species (*P*. *polyxenes*); substantial variability was present, however, particularly in *P*. *machaon*. The putative hybrids showed less within-species variability when compared to the parental species, although smaller sample sizes likely contributed to this pattern. *Papilio brevicauda* and *P*. *joanae* grouped closely with *P*. *polyxenes*, which is unsurprising based on their overall appearance (Fig 1). *Papilio machaon kahli* was found on the periphery of the *P*. *polyxenes* cluster, extending towards *P*. *zelicaon* & *P*. *machaon*. Finally, *P*. *zelicaon* x *machaon* hybrids grouped closely within the *P*. *zelicaon*/*P*. *machaon* cluster, with one black morph (“nitra”) individual extending towards the *P*. *polyxenes* region. Several individuals of *P*. *machaon* and *P*. *polyxenes* also occupy the intermediate space between the main groupings of their respective taxa (four *P*. *machaon* individuals to the left of x = -0.25, and three *P*. *polyxenes* individuals to the right of x = -0.4), and correspond to black morph *P*. *machaon bairdii* and yellow morph *P*. *polyxenes americus*.

### Molecular dating

Molecular dating of the complete COI/COII data set resulted in a root age for the divergence of *P*. *xuthus* from the remaining species of 18.25 (15.29–23.38 95% confidence interval) million years (MY) before present (Fig 2, Table 4), which, as expected, is quite similar to the age of the respective node found by [38]. This leads to the age of divergence of the main hybrid haplotype clade of 1.60 (0.24–2.45) MY, or in the mid-Pleistocene. The overall topology of this analysis was consistent with that obtained from MrBayes, except the monophyly of the main hybrid group was further broken by the presence of two *P*. *machaon* haplotypes (Fig. A in S1 File). Age estimates for main nodes in Fig 2 are provided in Table 4, and the entire dated tree is shown in Fig. A in S1 File.

**Table 4:** Age estimates and 95% confidence intervals of major nodes shown in Fig 2. All ages in millions of years.

Clade #	Age estimate	95% CI
1	18.25	15.29–23.38
2	7.07	5.02–9.01
3	5.12	3.77–7.01
4	1.60	0.24–2.45
5	3.46	0.51–6.71
6	1.51	0.08–3.73
7	1.46	0.07–3.67

## Discussion

Interspecific hybridization across the *P*. *machaon* species group in North America is supported by several mutually-reinforcing new lines of evidence. Using mtDNA sequence data, we identified a *machaon*-like lineage shared by four putative hybrid populations or species, confirming earlier findings based on mtDNA restriction-site variation [28]. In contrast, nuclear markers and morphological characters exhibit variable admixture and intermediacy, ranging from signatures indistinguishable from *P*. *polyxenes* or *P*. *zelicaon* to those of a stable hybrid swarm. The one nuclear gene that was sequenced, EF-1α, showed only shared sequence variation among the major species in the group, although it confirmed *P*. *indra* as the closest outgroup. Hybridization among species would have been facilitated by the repeated glaciations of the Pleistocene, and illustrates the importance that hybridization can have in the evolutionary histories of entire species groups. We first discuss this phylogeographic hypothesis, and then consider the relative roles of hybrid speciation and adaptive introgression in the formation of each of these hybrid lineages.

### Pleistocene origins and phylogeography of hybrid lineages

**Fig 6:**
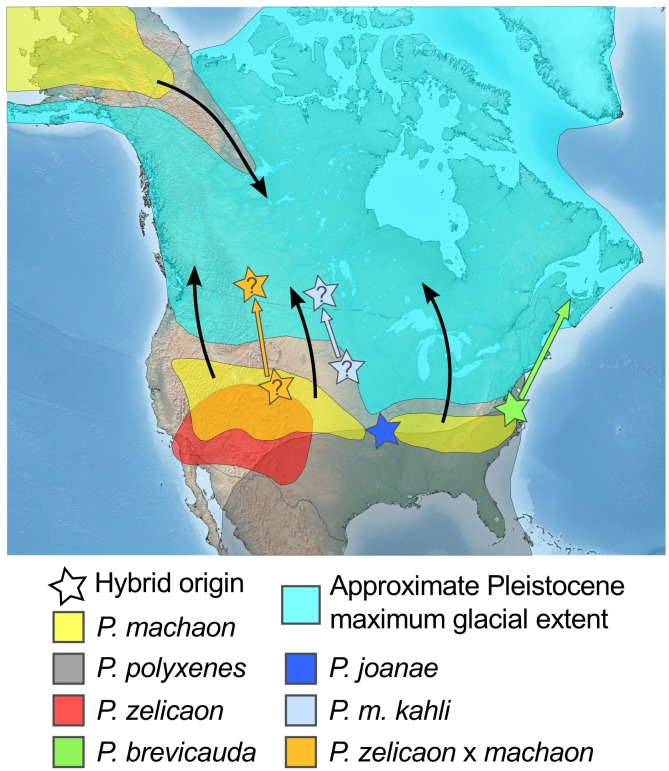
Approximate maximum glacial extent during the early- to mid-Pleistocene, hypothesized glacial refugia and hybrid origins. Arrows indicate hypothesized postglacial recolonization routes of hybrids (colored arrows), and parental species (black arrows). Hybrid origins with question marks indicate origins with less geographic certainty. Map image: public domain from www.simplemappr.net.

Molecular dating of COI/COII approximates the time of divergence of the main hybrid lineage as mid Pleistocene (Fig 2, Table 4). The use of a single marker and secondary calibrations make this a rough estimation (e.g. [89] but see [90] regarding the use of multiple secondary calibration points). Nonetheless, with confidence intervals of 1.1–2.8 MY, the initial hybridization events for all hybrid lineages can be confidently placed in the context of the repeated glaciations of the Pleistocene [91]. As [28] hypothesize, at these glacial maxima, the ranges of all three parental taxa would have been forced south, creating new regions of contact or sympatry between *P*. *machaon* and *P*. *polyxenes*/*P*. *zelicaon*, and facilitating hybridization (Fig 6). Subsequent glacial contraction likely separated sympatric populations, exposing hybrid remnants to differential introgression from their parental species.

*Papilio machaon hudsonianus* is the most easterly-distributed subspecies of *P*. *machaon* in North America [43] (Fig 1). The fact that *P*. *m*. *hudsonianus* mtDNA haplotypes (haplotype mach8) belong to the same mtDNA clade as the putative hybrid taxa supports a previously more widespread distribution for this mtDNA lineage. During or shortly after glacial maxima, *P*. *m*. *hudsonianus* from eastern North America would have been likely to come into contact with *P*. *polyxenes* in or near the current ranges of *P*. *joanae* and *P*. *brevicauda* (Fig 6). Interestingly, one specimen of *P*. *m*. *pikei* also displayed hybrid clade mtDNA (haplotype hyb13), and the sister clade to the main hybrid clade was found in individuals of both *P*. *m*. *hudsonianus* and *P*. *m*. *aliaska* (clade mach2/3/8). *Papilio machaon pikei* and *P*. *m*. *aliaska* have ranges parapatric or sympatric to that of *P*. *m*. *hudsonianus* in western Canada [43, 49] (Fig 1), and some gene flow between these three subspecies may be expected. However, both *P*. *m*. *aliaska* and *P*. *m*. *pikei* more commonly have typical *machaon*-like mtDNA haplotypes (e.g. haplotypes mach11, mach12, mach19) rather than those from the hybrid clade, suggesting that the presence of the hybrid clade haplotypes is due to relatively recent mitochondrial gene flow from *P*. *m*. *hudsonianus* to *P*. *m*. *aliaska* and *P*. *m*. *pikei*.

Two individuals that were identified as *P*. *m*. *aliaska*, based on general appearance, flight period, and habitat, exhibited *zelicaon*-like signatures for both mtDNA and microsatellites. Morphologically, these individuals clustered closer to *P*. *zelicaon* x *machaon* individuals, but exhibited a very *P*. *m*. *aliaska*-like overall appearance. They were collected alongside many typical *P*. *m*. *aliaska* as well as several individuals exhibiting hybrid-like mtDNA at a locality at the southern edge of the range of *P*. *m*. *aliaska*. These variable hybrid signatures, all observed on the same day at the same locality, illustrate the scale of evolutionary complexity that is observed in this group. Hybrid-like and more divergent *P*. *machaon* mtDNA signatures could be the remnants of an ancestral *P*. *machaon* population lineage once widespread in central and eastern Canada, which took part in hybridization at several locations. More geographically comprehensive sampling of *P*. *m*. *hudsonianus* and *P*. *m*. *aliaska* from the entirety of their ranges would shed further light on this hypothesis.

Alternative explanations for similar, and in some cases identical, *machaon*-like mtDNA haplotypes found in geographically disjunct putative hybrids are not supported by our data. The likelihood of this repeated geographic pattern arising by neutral chance (i.e. genetic drift [92]) is low, and although incomplete lineage sorting can generate phylogenetic patterns that mimic introgression, it would not be expected to leave any appreciable phylogeographic signal [93, 94]. *Wolbachia*, a bacterial endosymbiont that can cause cytonuclear discordance (e.g. [95]), has not been detected in the species group (Dupuis *personal observation*). Finally, if neutral processes such as drift or founders’ effects were to fix introgressed haplotypes in putative hybrid populations, allelic (or haplotype) diversity would be expected to be low [96], which was not observed.

### Hybrid speciation in the *P*. *machaon* group

Our data supports hybrid origins for four more-or-less distinct populations of the *P*. *machaon* species group. These lineages arose from similar situations, namely mating between *P*. *machaon* (likely *P*. *m*. *hudsonianus*) and *P*. *polyxenes*/*P*. *zelicaon*, but interestingly this has produced different outcomes in their current genetic composition and ecological associations. This repetition provides a unique framework to compare the outcomes of hybridization with regard to hybrid speciation and adaptive introgression. Both processes require hybridization between distinct biological entities, and generally result in some kind of cytonuclear discordance. While the distinction between hybrid speciation and adaptive introgression may seem arbitrary outside of speciation theory, biodiversity estimation and conservation generally rely on species as a common unit of diversity. In this setting, it is important whether adaptive traits have introgressed from one species to another or two species have hybridized to create a third (e.g. [97, 98, 99]).

The key to distinguishing hybrid species from cases of adaptive introgression is to diagnose unique hybrid traits or combinations that facilitate reproductive isolation of hybrids from *both* parents [2]. Although genomic admixture and morphological intermediacy are conceptually intuitive indicators of potential hybridization, they can be difficult to tease apart from ancestral polymorphism and recent gene flow [13], and by themselves may not be enough to define a species as a homoploid hybrid [2]. This is especially true in cases of backcrossing to a single parent species [3], as would be expected with the *P*. *machaon* complex. Therefore, ecological evidence of reproductive isolation from both parents is often used in defining hybrid species (e.g. [100, 101]), although this isolation need not be absolute (e.g. [102, 34, 103]).

**Fig 7:**
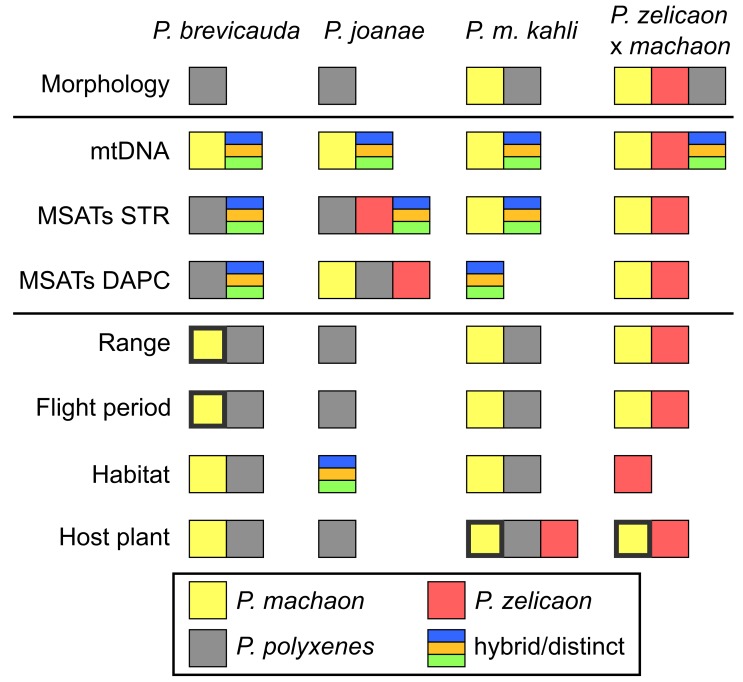
Summary of parental similarity and distinctiveness of four putative hybrid lineages with morphological, genetic, and ecological data. Colored boxes denote similarity or clustering with parental species, or distinctness of the hybrid lineage. Morphology is based on clustering in morphological analysis (MCA). mtDNA is based on mitochondrial DNA clades (note that the unique hybrid clade is identified as both distinct and as *P*. *machaon*-like). MSATs STR is based on summarized ancestry at the finest subpopulation scale in STRUCTURE analyses, and MSATs DAPC on summarized clustering in DAPC analyses. Range shows sympatry or parapatry with parental species, flight period shows overlapping adult flight period, habitat is based on a general assessment of shared habitat type (forest openings, strict hilltopping behavior, under forest cover, etc.), and host plant shows shared larval host. Boxes with wide outlines indicate uncertainty in particular characteristics. Ecological information is presented in more detail in Table D in S1 File.

Here we focus on two criteria for defining a lineage as a hybrid species: 1) a hybrid species must have shared characteristics with both parents, but also some level of distinctiveness that facilitates diagnosis of the hybrids; and more importantly, 2) a hybrid species must have some novel characteristics that facilitate reproductive isolation from the parental species [2]. For the purposes of this study, meeting both of these criteria provides support for the lineage in question being a hybrid species; while meeting only one criteria (e.g. displaying genealogical discordance or introgression, but no characteristics of reproductive isolation) is insufficient evidence, and is more likely a scenario involving adaptive introgression in the history of the lineage. Although this framework greatly simplifies a conceptually complicated and difficult task [2], it is appropriate given the nature of this genetic data (non-genomic) and the paucity of ecological information for these butterflies, which are often difficult to find. To assist in this evaluation, we have compiled pertinent ecological traits known to facilitate reproductive isolation in Table D in S1 File, and have summarized this information, as well as our morphological and genetic conclusions, in Fig 7.

#### Papilio brevicauda

To lepidopterists familiar with swallowtails, *P*. *brevicauda* is a clearly diagnosable entity: its combination of *polyxenes*-like (black morph, orange undersides of hind wings) and *machaon*-like traits (short hindwing tails and shorter, rounded forewings) is distinctive and supports its widely-recognized species status. Only the *polyxenes*-like characters were included in our morphological analysis, due to the limited quality of voucher specimen wings and subsequent missing data, which can account for its indeterminate placement in the MCA. Genetically, *P*. *brevicauda* also displays characteristics of both *P*. *machaon* and *P*. *polyxenes*, as well as some degree of distinctiveness in nuclear markers at a fine-scale, supporting its consideration as a hybrid species. However, it appears to have no clear ecological separation from either parental species that would provide reproductive isolation from them (Fig 7) [43, 42, 104, 105], although one unknown in this ecological assessment is the eastern range limit of *P*. *m*. *hudsonianus*. Historic and potentially ongoing introgression of *P*. *m*. *hudsonianus* genes into a northern-adapted, coastal lineage of *P*. *polyxenes* could account for *P*. *brevicauda*’s morphological and genetic intermediacy, but it requires that that *machaon*-like mtDNA experienced a selective sweep through the population [106]. However, with the data at hand, the evidence for novelty of putative hybrid characteristics or reproductive isolation is not substantial, and so *P*. *brevicauda* does not fully qualify as a hybrid species. Comprehensive geographic sampling, particularly at the western and southern edges of the range of *P*. *brevicauda* (where it may be sympatric or parapatric with *P*. *m*. *hudsonianus* and *P*. *polyxenes*, respectively), should clarify its status.

#### Papilio joanae

Unlike *P*. *brevicauda*, *P*. *joanae* is often morphologically indistinguishable from *P*. *polyxenes asterius* [43, 45], and has only a handful of *machaon*-like traits. Except for mtDNA, its genetic characteristics are more *polyxenes*-like, although at fine scales it is somewhat *zelicaon*-like (Figs 3 and 4). Many of *P*. *joanae*’s ecological traits are shared with *P*. *p*. *asterius* [107, 43], except for its affinity for closed forests and cedar glades [107], rather than the open habitats (fields, exposed hilltops) that are used by the rest of the species group. This strict use of forest habitats nonetheless provides substantial separation of the two species, as *P*. *joanae* larvae are only found on hosts within forest habitats and *P*. *p*. *asterius* larvae are found only in open areas (Heitzman *personal communication*). Interestingly, *P*. *m*. *hudsonianus* is the only other North American member of the species group that frequents forest edges and shaded habitats, and even oviposits in shaded areas (Dupuis *personal observation*), although it uses different hosts than *P*. *joanae*.

The novelty of this *P*. *m*. *hudsonianus*-like ecological characteristic, which contributes to reproductive isolation of *P*. *joanae* from *P*. *polyxenes*, supports the hypothesis that *P*. *joanae* is indeed a hybrid species. This isolation is similar to ecological separation in several other North American hybrid butterfly species [100, 34]. Adaptive introgression of *machaon*-like genes into *P*. *polyxenes* is also possible, but this alternative explanation is less likely. The nearest populations with similar mtDNA haplotypes are now over 1000 kilometers to the north. At finer scales, *P*. *joanae* displays *zelicaon*-like as well as *polyxenes*-like nuclear characteristics (Figs 3 and 4), suggesting older hybridization between *P*. *m*. *hudsonianus* and the common ancestor of *P*. *polyxenes* and *P*. *zelicaon*. For these reasons, we conclude that *P*. *joanae* is a homoploid hybrid species that is reproductively isolated from its parents via behavioral separation. This may be an important consideration for future conservation prioritization of *P*. *joanae* [45].

#### Papilio machaon kahli

*Papilio machaon kahli* is the most enigmatic of the putative hybrids considered here. Morphologically, it is intermediate between *P*. *machaon* and *P*. *polyxenes* (Fig 5), and lepidopterists have found it difficult to distinguish it from *P*. *polyxenes* based on overall appearance [108, 42]. Genetically, both mtDNA and nuclear DNA show ties to *P*. *machaon* (Figs 2 and 3A), but at a finer scale *P*. *m*. *kahli* is quite distinct from the rest of the species group (Figs 3D and 4). However, more samples are needed to elaborate this potential unique signature. We find little ecological support for any traits that would provide reproductive isolation from *P*. *machaon* or *P*. *polyxenes*; the use of its main host, *Zizia aptera*, may provide separation from *P*. *m*. *hudsonianus*, although the range of hosts used by the latter subspecies is unclear [26, 48]. Interestingly, in the past 100 years there may have been a decline in the presence of *kahli*-like individuals throughout the small range of this taxon, and an increase in the presence of *P*. *p*. *asterius* [26]. Unfortunately our study includes only one specimen collected more recently than 1990 (Table A in S1 File), so we cannot attest to the current status of that trend, but microsatellite data clustering shows little sign of *polyxenes*-like ancestry. Overall, we find no support for hybrid species status, and, based on the data at hand, we consider it most likely that *P*. *m*. *kahli* is a transitional population of *P*. *m*. *hudsonianus* experiencing adaptive introgression from *P*. *polyxenes*. The geographically limited range of this lineage was likely instrumental in its taxonomic recognition as a subspecies.

#### *Papilio zelicaon* x *machaon*

Populations of *P*. *zelicaon* x *machaon* in SW Alberta display a very different scenario compared to other potential hybrid taxa, in that hybrids are parapatric with both parental taxa, and all three mtDNA types are found in the same populations. Nuclear admixture is also variable among individuals, resembling early-generation hybrids (F1, F2, backcross) and both *P*. *machaon* and *P*. *zelicaon*. Morphological variation in these populations mirrors the nuclear admixture, although black (“nitra”) morphs resembling *P*. *polyxenes* are also observed (Fig 7). Host plants and habitat preferences may provide hybrids with some ecological separation from *P*. *m*. *dodi*, which feeds strictly on *Artemesia dracunculus* in arid river valleys, but not from *P*. *zelicaon* [26]; adult hilltopping locations of *P*. *m*. *dodi* and *P*. *zelicaon* are often close enough to each other that both species can be found in the other’s respective habitat. The southern range limit of *P*. *m*. *hudsonianus* is unclear, although *hudsonianus*-like specimens have been observed in southern Alberta in the vicinity of hybrid populations [49]. Whether these rare occurrences represent migrants from farther north or persisting populations is unknown. If they do represent the latter, then host choice may also foster ecological isolation between *P*. *m*. *hudsonianus* and *P*. *zelicaon* x *machaon* individuals.

The presence of mitochondrial, nuclear, and morphological intermediates at varying stages of evolutionary separation indicates that these populations represent a stable hybrid swarm [4, 109], and are far from being a distinct hybrid species. Predominant *zelicaon*-like ancestry is indicated with nuclear markers (Figs 3 and 4), but more comprehensive geographic sampling would clarify the situation. Interestingly, morphologically similar intermediates have been collected from the area since the early twentieth century before significant agricultural habitat changes [26], indicating that there was no anthropogenic influence on the initial formation of a hybrid population. Whether this situation will progress to hybrid speciation, as seen with *P*. *joanae*, will depend on the development of mechanisms for ecological and reproductive isolation from both parental species.

### Other lepidopteran hybrids

New World Lepidoptera have had disproportionate influence on the study of hybridization, due in part to their high diversity and general appeal, and these systems provide a rich foundation to compare to that of the *P*. *machaon* group. *Lycaeides* butterflies in western North America share many similarities with the *P*. *machaon* group, particularly in the multifarious nature of hybridization observed in the group [110]. Widespread historic admixture throughout *Lycaeides* is reminiscent of the *P*. *m*. *hudsonianus*-like signatures observed across North America in putative hybrid lineages. Additionally, ecological separation from parental taxa has allowed some hybrids to persist in novel habitats, akin to *P*. *joanae* [100]. *Heliconius* butterflies in Central and South America regularly hybridize (e.g. [111, 112, 113]), similarly to members of the *P*. *machaon* complex, and abundant ecological and genomic data has revealed promiscuous exchange of genes controlling protective color-pattern between hybridizing species [17]. However, contention regarding the hybrid origins of some *Heliconius* species [114] emphasizes the importance of addressing the fine differences between hybrid speciation and adaptive introgression. Within the Papilionidae, ecological and genomic data support the hybrid status of *Papilio appalachiensis* [33, 35, 115], where it is also clear that ecological separation has aided reproductive isolation from the parental species [34], again similarly to *P*. *joanae*. With growing insight into the potential for hybridization to encourage diversification and adaptation (e.g. [110, 17]), we are confident that continued work on the *P*. *machaon* group will facilitate this understanding, and support [46] assertion “that the Machaon-group provides some of the most suitable material ever investigated in animals for studying the process of speciation in detail.”

## Conclusions

Our results demonstrate a case of repeated reticulate evolution within a species complex of swallowtail butterflies in North America. We have documented three geographically separated cases of cytonuclear discordance where mtDNA is completely fixed in hybrid lineages (*P*. *brevicauda*, *P*. *joanae*, and *P*. *m*. *kahli*), and one case where both hybrid and parental mitochondrial haplotypes occur (*P*. *zelicaon* x *machaon*). Excluding the parental-like mtDNA haplotypes found in *P*. *zelicaon* x *machaon* populations, all of the mtDNA of hybrids is likely derived from a single *machaon*-like lineage resembling *P*. *m*. *hudsonianus*, despite geographic separation of these populations from *P*. *machaon* and from each other. Nuclear markers show variable signatures ranging from almost completely paternal characteristics, to high levels of admixture and potentially unique hybrid signatures; morphological characters also show variable levels of intermediacy. The divergences of these hybrid lineages were dated to the mid-Pleistocene, indicating an important role for glacial refugia in their formation. Despite similar hybrid origins (*P*. *machaon* hybridizing with *P*. *polyxenes*/*P*. *zelicaon*), these lineages have followed distinct evolutionary trajectories leading to diverse outcomes, from hybrid speciation (*P*. *joanae*) and potential adaptive introgression (*P*. *brevicauda* and *P*. *m*. *kahli*) to stable hybrid swarms (*P*. *zelicaon* x *machaon*). These results add to a growing recognition of the evolutionary importance and complexity of hybridization in generating biodiversity.

## Supporting Information

S1 File Supplementary file. Contains: Fig. A in S1 File. Molecular dated tree based on COI/COII data secondarily calibrated with four nodes; Fig. B in S1 File. STRUCTURE results for all microsatellite data, including *P*. *indra*; Fig. C in S1 File. STRUCTURE results for microsatellite genotype data for the overall dataset, including substructure; Fig. D in S1 File. DAPC for all microsatellite data, including *P*.*indra*; Fig. E in S1 File. Morphometric MCA without using the *jitter* function to separate overlapping points; Table A in S1 File. Specimen information; Table B in S1 File. Microsatellite loci used in this study; Table C in S1 File. Primers for new sequences used in this study; and, Table D in S1 File. Summary of morphological and ecological information pertinent to the species included in this study.
(PDF)

S2 File Microsatellite data. (STR)

S3 File Morphological data. (TXT)
