# Supplementary material for: Repeated Reticulate Evolution in North American Papilio machaon Group Swallowtail Butterflies
Source: PLoS One. 2015 Oct 30;10(10):e0141882. doi: 10.1371/journal.pone.0141882 (PMC4627828; doi:10.1371/journal.pone.0141882)
Supplement: S1 File — Contains: Fig. A in S1 File. Molecular dated tree based on COI/COII data secondarily calibrated with four nodes; Fig. B in S1 File. STRUCTURE results for all microsatellite data, including P. indra; Fig. C in S1 File. STRUCTURE results for microsatellite genotype data for the overall dataset, including substructure; Fig. D in S1 File. DAPC for all microsatellite data, including P.indra; Fig. E in S1 File. Morphometric MCA without using the jitter function to separate overlapping points; Table A in S1 File. Specimen information; Table B in S1 File. Microsatellite loci used in this study; Table C in S1 File. Primers for new sequences used in this study; and, Table D in S1 File. Summary of morphological and ecological information pertinent to the species included in this study. (PDF) [file pone.0141882.s001.pdf]

S1 File: Supporting Information

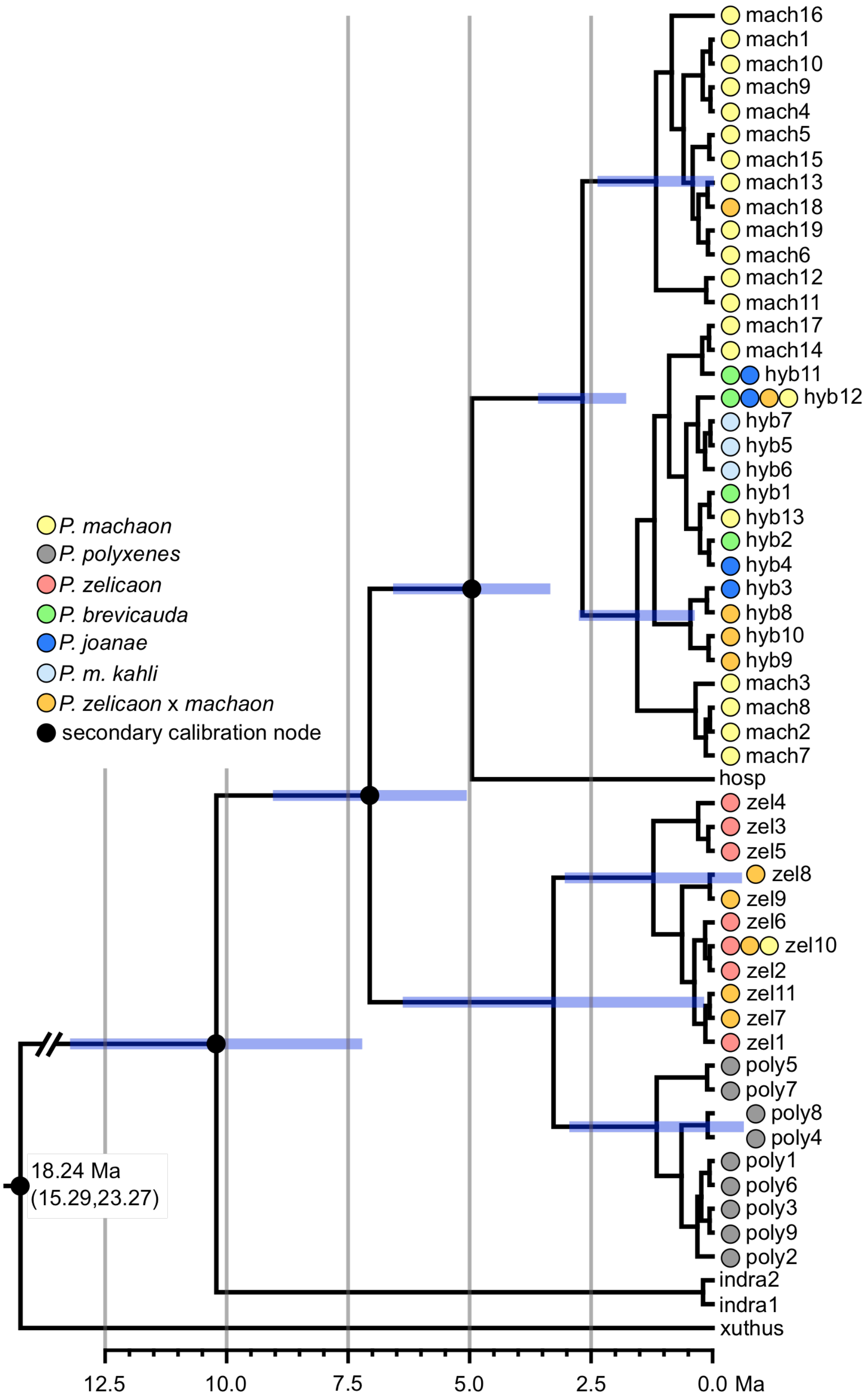

Fig. A. **Molecular dated tree based on COI/COII data secondarily calibrated with four nodes.**

95% confidence intervals shown with blue bars.

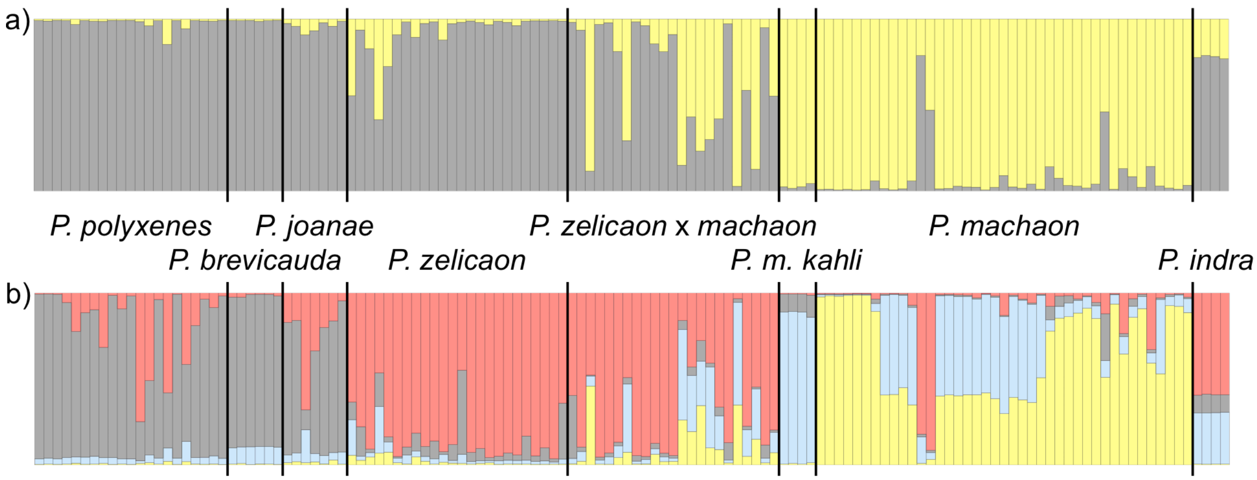

Fig. B. **STRUCTURE results for all microsatellite data, including *P. indra*.** a) $k = 2$, and b) $k = 4$.

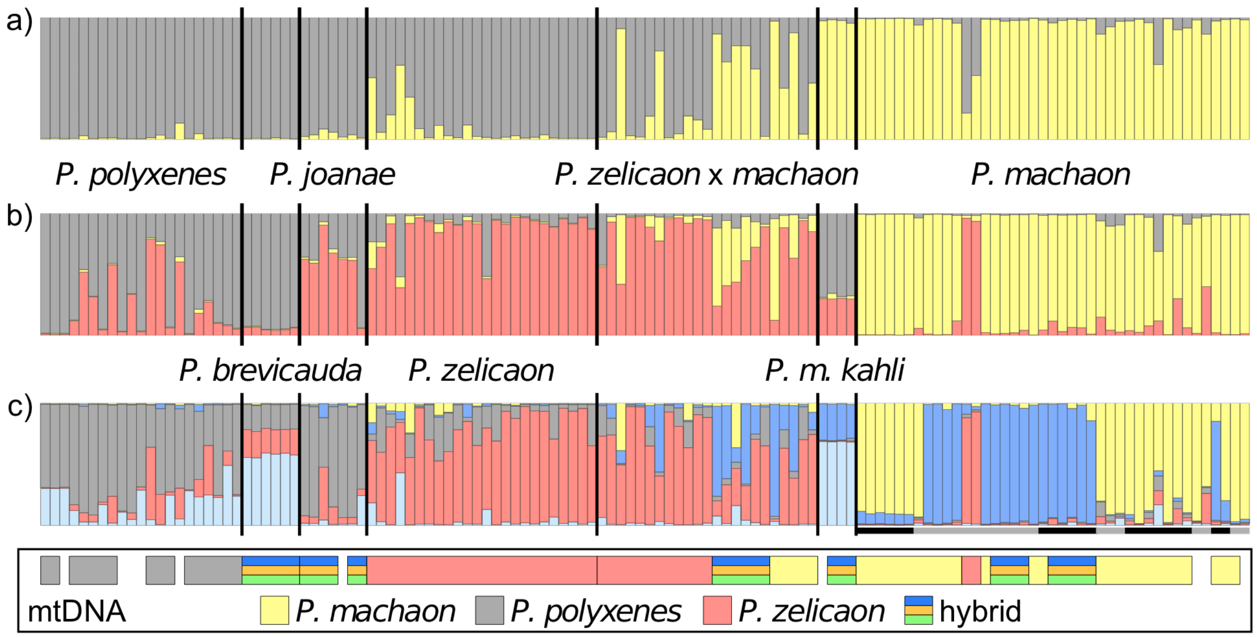

Fig. C. **STRUCTURE results for microsatellite genotype data for the overall dataset, including substructure**. a) $k = 2$ (identical to Figure 3), b) $k = 3$, and c) $k = 5$. Inset indicates the major mtDNA clade (*P. machaon*, *P. polyxenes*, *P. zelicaon*, or the main hybrid clade within the *P. machaon* clade) for each individual (gaps indicate specimens genotyped for microsatellites that were not sequenced for COI/COII). Alternating black and grey bars below the *P. machaon* portion of c) indicate subspecies;

from left to right: *P. m. pikei*, *aliaska*, *hudsonianus*, *bairdii*, *dodi*, *oregonius*, *gorganus*, and *hippocrates*.

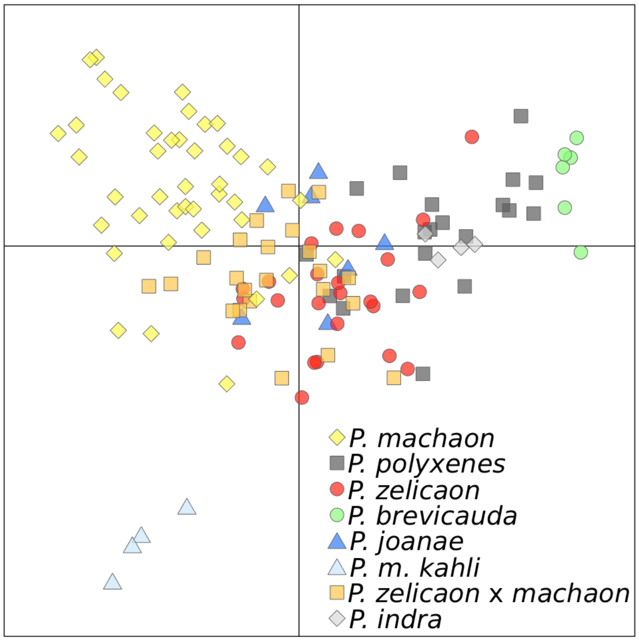

Fig. D. **DAPC for all microsatellite data, including *P.indra*.**

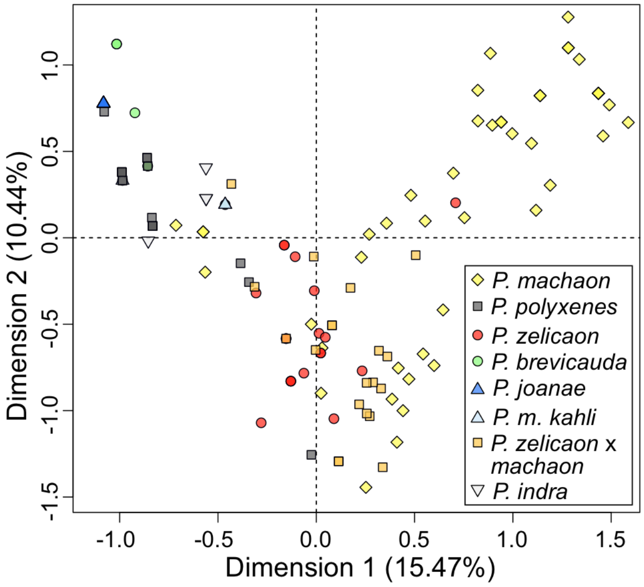

Fig. E. **Morphometric MCA without using the *jitter* function to separate overlapping points.**

Table A. **Specimen information.** COI/COII and EF-1α columns provide GenBank accession numbers followed by the haplotype names used in the present study; for COI/COII, haplotype names in parentheses are those used in Sperling & Harrison (1994) (Some specimens were used in Sperling & Harrison (1994), but were not sequenced for this study). Italicized GenBank accession numbers represent sequences not generated in this study. Asterisks in columns S and M indicate specimens used for microsatellite and morphometric analyses, respectively.

#	Species	Locality; collector; year collected; host (if available); latitude longitude (if available)	COI/COII	EF-1α	S	M
FS223.h15	*P. machaon aliaska*	USA: Alaska: Eagle Summit; Ayres, M.P.; 1990; 65.48453 -145.40346	*FJ808909.1* mach3			
FS260	*P. machaon aliaska*	Canada: British Columbia: Alaska Hwy: Pink Mtn; Troubridge, J.T.; 1990; 57.05197 -122.86839	KJ363206 mach2(M11)	KJ363312 mach2	*	*
JRD136	*P. machaon aliaska*	Canada: British Columbia: Alaska Hwy: Pink Mtn; Dupuis, J.R.; 2010; 57.05197 -122.86839	KJ363246 mach19		*	*
JRD139	*P. machaon aliaska*	Canada: British Columbia: Alaska Hwy: Pink Mtn; Dupuis, J.R.; 2010; 57.05197 -122.86839	KJ363247 mach19		*	*
JRD150	*P. machaon aliaska*	Canada: British Columbia: Alaska Hwy: Pink Mtn; Dupuis, J.R.; 2010; 57.05197 -122.86839	KJ363249 mach19		*	*
JRD152	*P. machaon aliaska*	Canada: British Columbia: Alaska Hwy: Pink Mtn; Dupuis, J.R.; 2010; 57.05197 -122.86839	KJ363248 mach19		*	*
JRD156	*P. machaon aliaska*	Canada: British Columbia: Alaska Hwy: Pink Mtn; Dupuis, J.R.; 2010; 57.05197 -122.86839	KJ363250 hyb12		*	*
JRD158	*P. machaon aliaska*	Canada: British Columbia: Alaska Hwy: Pink Mtn; Dupuis, J.R.; 2010; 57.05197 -122.86839	KJ363253 zel10		*	*
JRD164	*P. machaon aliaska*	Canada: British Columbia: Alaska Hwy: Pink Mtn; Dupuis, J.R.; 2010; 57.05197 -122.86839	KJ363257 zel10		*	*
JRD169	*P. machaon aliaska*	Canada: British Columbia: Alaska Hwy: Pink Mtn; Dupuis, J.R.; 2010; 57.05197 -122.86839	KP262869 hyb12		*	*
JRD415	*P. machaon aliaska*	Canada: British Columbia: Alaska Hwy: Pink Mtn; Sperling, F.A.H.; 1998; 57.05197 -122.86839	KP262873 mach19		*	*
JRD669	*P. machaon aliaska*	Canada: British Columbia: Alaska Hwy: Pink Mtn; Schmidt, B.C.; 2005; 57.05197 -122.86839	KP262874 hyb12		*	*

JRD670	*P. machaon aliaska*	Canada: British Columbia: Alaska Hwy: Pink Mtn; Schmidt, B.C.; 2005; 57.05197 -122.86839	KP262875 hyb12		*	*
FS176	*P. machaon bairdii*	USA: California: San Bernardino co.: Sugarloaf Mtn.; Emmel, J.F.; 1990; 34.19918 -116.81460	KJ363223 mach4(M3)			*
FS188	*P. machaon bairdii*	USA: California: San Bernardino co.: Sugarloaf Mtn.; Emmel, J.F.; 1990; 34.19918 -116.81460	KJ363224 mach4(M3)			*
FS194	*P. machaon bairdii*	USA: Arizona: Apache co.: Eagar; McCorkle, D.V.; 1989	KJ363225 mach19(M4)			*
FS211	*P. machaon bairdii*	USA: California: San Bernardino co.: Sugarloaf Mtn.; Emmel, J.F.; 1990; 34.19918 -116.81460	KJ363231 mach19(M3)		*	*
FS204	*P. machaon bairdii (brucei)*	USA: Nebraska: west Nebraska: Dawson co.; Spomer, S. via Heitzman, J.R.; 1990	KJ363203 mach1(M5)	KJ363309 mach1	*	*
FS265	*P. machaon bairdii(brucei)*	USA: Nebraska: west Nebraska; Spomer, S. via Heitzman, J.R.; 1990	KJ363234 mach19(M2)		*	*
FS277	*P. machaon bairdii(brucei)*	USA: Colorado: Freemont co.; Fisher, M.S.; 1990	KJ363235 mach19(M3)		*	*
FS155	*P. machaon dodi*	USA: Montana: Circle; Sperling, F.A.H.; 1987	KJ363219 mach6(M2)		*	*
JRD209	*P. machaon dodi*	Canada: Alberta: Drumheller: Tolman Bridge; Dupuis, J.R.; 2010; 51.84258 -113.00796	KJ363258 mach5		*	*
JRD210	*P. machaon dodi*	Canada: Alberta: Drumheller: Tolman Bridge; Dupuis, J.R.; 2010; 51.84258 -113.00796	KJ363259 mach5		*	*
JRD211	*P. machaon dodi*	Canada: Alberta: Drumheller: Tolman Bridge; Dupuis, J.R.; 2010; 51.84258 -113.00796	KJ363260 mach5		*	
JRD223	*P. machaon dodi*	Canada: Alberta: Drumheller: Tolman Bridge; Dupuis, J.R.; 2010; 51.84258 -113.00796	KJ363261 mach5		*	*
JRD224	*P. machaon dodi*	Canada: Alberta: Drumheller: Tolman Bridge; Dupuis, J.R.; 2010; 51.84258 -113.00796	KJ363262 mach5		*	*
JRD227	*P. machaon dodi*	Canada: Alberta: Drumheller: Tolman Bridge; Dupuis, J.R.; 2010; 51.84258 -113.00796	KJ363263 mach5		*	*
FS027	*P. machaon gorganus*	France: Coudoux; Piquemal L., via Hauser, C.; 1987	*AF044006.1* mach15(M1)	AF044819.1 mach15	*	*
FS156	*P. machaon gorganus*	Czech Republic: Prague; Häuser, C.; 1987	KJ363220 mach16(M3)		*	*
FS078	*P. machaon hippocrates*	Japan: Gifu Pref.: Vicinity of Gifu; Okura, J.; 1989	*AY457593.1* mach14(M8)	*AY457621.1* mach14	*	*
FS323	*P. machaon hippocrates*	Japan: Tokyo Prefecture: Mount Takao; West, D.A.; 1990	KJ363241 mach17(M8)			*
FS431	*P. machaon hippocrates*	Japan: Aichi Prefecture: Nagoya; Ae, S.A.; 1990	(M8)		*	*

JRDB073	*P. machaon hudsonianus*	Canada: Manitoba: N of Duck Mtn.; Dupuis, J.R.; 2014; 51.9944 -101.0353	KP262876 hyb12		*	*
JRDB074	*P. machaon hudsonianus*	Canada: Manitoba: N of Duck Mtn.; Dupuis, J.R.; 2014; 51.9944 -101.0353	KP262877 mach8		*	*
JRDB076	*P. machaon hudsonianus*	Canada: Manitoba: N of Duck Mtn.; Dupuis, J.R.; 2014; 51.9944 -101.0353	KP262878 hyb12		*	*
JRDB077	*P. machaon hudsonianus*	Canada: Manitoba: N of Duck Mtn.; Dupuis, J.R.; 2014; 51.9944 -101.0353	KP262879 hyb12		*	*
JRDB078	*P. machaon hudsonianus*	Canada: Manitoba: N of Duck Mtn.; Dupuis, J.R.; 2014; 51.9944 -101.0353	KP262880 hyb12		*	*
JRDB079	*P. machaon hudsonianus*	Canada: Manitoba: N of Duck Mtn.; Dupuis, J.R.; 2014; 51.9944 -101.0353	KP262881 hyb12		*	*
JRDB098	*P. machaon hudsonianus*	Canada: Québec: E of Chisasibi: salt marsh; Larrivee, M.; 2010; on *Ligusticum scoticum*	KP262882 hyb12			
FS077	*P. machaon oregonius*	USA: Washington: Palouse Falls; Anderson, S.S.; 1988; 46.656509 -118.228575	*AF044007.1* mach9(M3)	*AF044828.2* mach9		*
FS234	*P. machaon oregonius*	USA: Washington: Klickitat co.; McCorkle, D.V.; 1990	(M3)		*	*
FS377	*P. machaon oregonius*	USA: Washington: Wishram: on Columbia River; McCorkle, D.V.; 1986	(M3)		*	*
2300.h2	*P. machaon pikei*	Canada: Alberta: Peace River: S of Bear Canyon; Baker, A. & Szkoropa, T.; 2002; 56.16706 -119.81085	*FJ808896.1* mach13			
2804.h5	*P. machaon pikei*	Canada: Alberta: S of Peace River: Rd. to Judah; Bromilow S., Schmidt C.; 2005; 56.151 -117.315	*FJ808899.1* hyb13			
FS263	*P. machaon pikei*	Canada: British Columbia: Taylor; Troubridge, J.T.; 1990; 56.15403 -120.71828	KJ363207 mach12(M10)	KJ363313 mach12	*	*
JRD175	*P. machaon pikei*	Canada: Alberta: Peace River: Highland Park N; Dupuis, J.R.; 2010; 56.13076 -118.88931	KJ363251 mach11		*	*
JRD177	*P. machaon pikei*	Canada: Alberta: Peace River: Highland Park N; Dupuis, J.R.; 2010; 56.13076 -118.88931	KJ363252 mach11		*	*
JRD180	*P. machaon pikei*	Canada: Alberta: Peace River: Kaufman Hill; Dupuis, J.R.; 2010; 56.24883 -117.27408	KJ363254 mach19		*	*
JRD181	*P. machaon pikei*	Canada: Alberta: Peace River: Kaufman Hill; Dupuis, J.R.; 2010; 56.24883 -117.27408	KJ363255 mach19		*	*
JRD182	*P. machaon pikei*	Canada: Alberta: Peace River: Kaufman Hill; Dupuis, J.R.; 2010; 56.24883 -117.27408	KJ363256 mach11		*	*

FS082b	*P. polyxenes americus*	Ecuador: Quito; Ponce, P. via Emmel, J.F.; 1989	KJ363196 poly1(P2)	KJ363302 poly1	*	*
FS102	*P. polyxenes americus*	Ecuador: Quito; Ponce, P. via Emmel, J.F.; 1989	KJ363216 poly9(P2)		*	*
FS110	*P. polyxenes americus*	Ecuador: Napo Prov.: Baños; Maudsley, J.R.; 1989	(P1)			*
FS274	*P. polyxenes americus*	Ecuador: Ambato; Levasseur, J. via McCorkle, D.V.; 1990	(P3)		*	*
FS013	*P. polyxenes asterius*	USA: New York: Watkins Glen; Sperling, F.A.H.; 1987; on *Pastinaca sativa*; 42.379 -76.871	KJ363211 poly2(P1)		*	*
FS064	*P. polyxenes asterius*	USA: New York: Ithaca: Feeny culture; Feeny, P.; 1989	*AF044010.1* poly3(P1)	*AF044823.2* poly3	*	*
FS087	*P. polyxenes asterius*	USA: Pennsylvannia: Pine Grove; Houtz, W.; 1989	KJ363215 poly9(P1)		*	
FS199	*P. polyxenes asterius*	USA: Missouri: Benton co.: Truman State Park; Heitzman, J.R.; 1990; 38.2687 -93.4463	KJ363227 poly9()		*	*
FS206	*P. polyxenes asterius*	USA: Michigan: Mackinac co.; Herig, T.; 1990	KJ363229 poly9(P1)		*	*
FS207	*P. polyxenes asterius*	Canada: Ontario: Port Hope: Rod Parrott culture; Parrot, R.; 1990	(P1)		*	*
FS221	*P. polyxenes asterius*	USA: Missouri: Lees Summit; Heitzman, J.R.; 1990; on parsley; 38.8988 -94.3832	(P1)		*	*
FS317	*P. polyxenes asterius*	USA: Missouri: Independence; Heitzman, J.R.; 1990; on *Foeniculum vulgare*; 39.1144 -94.4391	(P1)		*	*
JRDB003	*P. polyxenes asterius*	USA: South Carolina: ; Zakharov, E.; 2002	KJ363296 poly9		*	*
JRDB004	*P. polyxenes asterius*	USA: North Carolina: Stanleyville: Ziglar Rd.; Sperling, F.A.H.; 2000; 36.19497 -80.28906	KJ363297 poly4		*	*
JRDB006	*P. polyxenes asterius*	USA: Pennsylvania: N of Ligonier: Wineland Rd.; Hilchie, G.J.; 2012; 40.2905 -79.2019	KJ363270 poly9		*	
FS080	*P. polyxenes coloro*	USA: California: Hemet; Emmel, J.F.; 1989; on *Ruta graveolens*	(P1)			*
FS177	*P. polyxenes coloro*	USA: California: San Diego co.: Jacumba; Griffin, B. via Emmel, J.F.; 1989; 32.61617 -116.18943	(P1)		*	*
FS191	*P. polyxenes coloro*	USA: Arizona: Maricopa co.: Four Peaks Rd.; Griffin, B. via Emmel, J.F.; 1989; 33.66964 -111.49151	KJ363201 poly5(P1)	KJ363307 poly5	*	*
FS218	*P. polyxenes coloro*	USA: California: Riverside co.: Hemet; Emmel, J.F.; 1990; on *Ruta graveolens*; 33.7203 -116.9343	KJ363232 poly9(P1)		*	*
JRDB009	*P. polyxenes coloro*	USA: Arizona: Portal: AMNH SW Research Station; Anweiler, G.; 2012; 31.912676 -109.141157	KJ363298 poly6		*	*

JRDB010	*P. polyxenes coloro*	USA: Arizona: Portal: AMNH SW Research Station; Anweiler, G.; 2012; 31.912676 -109.141157	KJ363299 poly7		*	*
JRDB012	*P. polyxenes coloro*	USA: Arizona: Portal: AMNH SW Research Station; Anweiler, G.; 2012; 31.912676 -109.141157	KJ363271 poly9		*	
FS220	*P. polyxenes stabilis*	Costa Rica: Monteverde; Joyce, F.; 1990; on *Anethum graveolens*	KJ363204 poly8(P1)	KJ363310 poly8	*	*
FS051	*P. zelicaon*	USA: California: Hemet; Emmel, J.F.; 1989; 33.7203 -116.9343	KJ363213 zel10(Z1)		*	*
FS052	*P. zelicaon*	USA: Washington: Juniper Dunes; Wehling, W.; 1989; 46.38883 -118.85631	KJ363214 zel10(Z1)		*	*
FS060	*P. zelicaon*	USA: South Dakota: Black Hills: near Silver City; Sperling, F.A.H.; 1987; 44.07693 -103.5725	KJ363195 zel4(Z5)	KJ363301 zel4		
FS076	*P. zelicaon*	USA: California: Riverside co.: Hemet; Emmel, J.F.; 1989; 33.7203 -116.9343	*AF044008.1* zel5(Z1)	*AF044827.1* zel5	*	*
FS153	*P. zelicaon*	USA: South Dakota: Black Hills: near Silver City; Sperling, F.A.H.; 1987; on *Zizia aptera*; 44.07693 -103.5725	KJ363218 zel10(Z4)		*	*
FS161	*P. zelicaon*	USA: South Dakota: Black Hills: near Silver City; Sperling, F.A.H.; 1987; 44.07693 -103.5725	KJ363222 zel2(Z6)		*	*
FS165	*P. zelicaon*	USA: California: Hemet; Emmel, J.F.; 1990; on *Foeniculum vulgare*; 33.7203 -116.9343	KJ363273 zel10()		*	*
FS174	*P. zelicaon*	USA: South Dakota: Black Hills: near Silver City; Sperling, F.A.H.; 1987; 44.07693 -103.5725	KJ363274 zel10(Z4)		*	*
FS182	*P. zelicaon*	USA: Colorado: Jefferson co.: Lookout Mtn.; Fisher, M.S.; 1990; 39.7336 -105.2380	KJ363199 zel1(Z6)	KJ363305 zel1_1	*	
FS183	*P. zelicaon*	USA: Colorado: Jefferson co.: Lookout Mtn.; Fisher, M.S.; 1990; 39.7336 -105.2380	KJ363275 zel2(Z6)		*	*
FS186	*P. zelicaon*	USA: Washington: Colockum Pass Rd.; Peterson, M.A.; 1990; 47.2608 -120.1873	KJ363276 zel10(Z1)		*	*
FS187	*P. zelicaon*	USA: Washington: Wilson Creek Rd.; Peterson, M.A.; 1990; 47.0874 -120.4981	KJ363200 zel1(Z4)	KJ363306 zel1_2	*	*
FS208	*P. zelicaon*	USA: Colorado: Jefferson co.: Lookout Mtn.; Fisher, M.S.; 1990; 39.7336 -105.2380	KJ363230 zel6(Z4)		*	*
FS229	*P. zelicaon*	USA: South Dakota: Black Hills: near Silver City; Sperling, F.A.H.; 1987; 44.07693 -103.5725	KJ363233 zel10(Z4)		*	*

FS284	*P. zelicaon*	USA: Washington: Columbia River: Rock Ck.; McCorkle, D.V.; 1990	KJ363237 zel3(Z1)		*	*
FS296	*P. zelicaon*	USA: Colorado: Jefferson co.: Lookout Mtn.; Fisher, M.S.; 1990; 39.7336 -105.2380	KJ363239 zel2(Z6)		*	*
FS393	*P. zelicaon*	Canada: British Columbia: Vancouver area; Troubridge, J.T.; 1990; 49.2 -122.8	KJ363243 zel2(Z4)		*	
FS394	*P. zelicaon*	Canada: British Columbia: Vancouver area; Troubridge, J.T.; 1990; 49.2 -122.8	KJ363244 zel3(Z1)		*	
FS420	*P. zelicaon*	USA: California: Hemet; Emmel, J.F.; 1989; 33.7203 -116.9343	KJ363245 zel2(Z4)		*	
JRD295	*P. zelicaon*	Canada: Alberta: Drumheller: Wintering Hills E; Dupuis, J.R.; 2011; 51.25993 -112.45478	KJ363264 zel2		*	*
JRD296	*P. zelicaon*	Canada: Alberta: Drumheller: Wintering Hills E; Dupuis, J.R.; 2011; 51.25993 -112.45478	KJ363265 zel2		*	*
JRD301	*P. zelicaon*	Canada: Alberta: Drumheller: Wintering Hills W; Dupuis, J.R.; 2011; 51.25520 -112.62614	KJ363266 zel10		*	
JRD302	*P. zelicaon*	Canada: Alberta: Drumheller: Wintering Hills W; Dupuis, J.R.; 2011; 51.25520 -112.62614	KJ363267 zel2		*	*
JRD303	*P. zelicaon*	Canada: Alberta: Drumheller: Wintering Hills E; Dupuis, J.R.; 2011; 51.25993 -112.45478	KJ363268 zel2		*	*
JRD306	*P. zelicaon*	Canada: Alberta: Drumheller: Wintering Hills E; Dupuis, J.R.; 2011; 51.25993 -112.45478	KJ363269 zel2		*	*
FS319	*P. brevicauda brevicauda*	Canada: Newfoundland: Fishell; Tremblay, N.; 1990	KJ363193 hyb1(M12)		*	*
FS320	*P. brevicauda brevicauda*	Canada: Newfoundland: Fishell; Tremblay, N.; 1990	KJ363240 hyb12(M12)		*	*
JRDB001	*P. brevicauda brevicauda*	Canada: Newfoundland: LaScie; Anweiler, G.; 2012; 49.9577 -55.6119	KJ363294 hyb2		*	*
JRDB002	*P. brevicauda brevicauda*	Canada: Newfoundland: LaScie; Anweiler, G.; 2012; 49.9577 -55.6119	KJ363295 hyb12		*	*
FS056	*P. brevicauda gaspeensis*	Canada: Quebec: Gaspe; Sperling, F.A.H.; 1988; on *Heracleum lanatum*	KJ363194 hyb11(M12)	KJ363300 hyb11_2		*
FS281	*P. brevicauda gaspeensis*	Canada: New Brunswick: Shippegan; McCloud, E. via Berenbaum, M.; 1990; on *Logusticum scoticum*	KJ363236 hyb12(M12)		*	*
FS321	*P. brevicauda gaspeensis*	Canada: Quebec: Gaspe; Tremblay, N.; 1990	KJ363210 hyb11(M12)	KJ363316 hyb11_3	*	*
FS120	*P. joanae*	USA: Missouri; Yoon, C. via McCorkle, D.V.; 1990	KJ363217 hyb12()		*	*
FS160	*P. joanae*	USA: Missouri; Yoon, C. via McCorkle, D.V.; 1990	KJ363221 hyb12()		*	*

FS198	*P. joanae*	USA: Missouri: Benton co.: 2.5 mi N of Truman State Park; Heitzman, J.R.; 1990; 38.31256 -93.44157	KJ363226 hyb12()		*	*
FS227	*P. joanae*	USA: Missouri: Benton co.: 2.5 mi N of Truman State Park; Heitzman, J.R.; 1990; 38.31256 -93.44157	KJ363205 hyb3(M12)	KJ363311 hyb3	*	*
FS288	*P. joanae*	USA: Missouri: Benton co.: 2.5 mi N of Truman State Park; Heitzman, J.R.; 1990; 38.31256 -93.44157	(M12)		*	*
FS311	*P. joanae*	USA: Missouri: C. Yoon culture from R. Heitzmann; Heitzman, J.R. via McCorkle, D.V.; 1986	KJ363209 hyb11(M13)	KJ363315 hyb11_1	*	*
FS312	*P. joanae*	USA: Missouri: Benton co.: W of Warsaw; Heitzman, J.R.; 1990; 38.24686 -93.40773	KJ363192 hyb4(M12)		*	*
FS279	*P. machaon kahli*	Canada: Manitoba: near Winnipeg; McCorkle, D.V.; 1990; 50.0014 -96.9167	()		*	
FS290	*P. machaon kahli*	Canada: Manitoba: Winnipeg: Bird Hill Park; McCorkle, D.V. via Hansen, K.; 1987; 50.0014 -96.9167	KJ363238 hyb5(M12)		*	*
FS328	*P. machaon kahli*	Canada: Manitoba: near Winnipeg; McCorkle, D.V.; 1990; 50.0014 -96.9167	KJ363242 hyb6()		*	*
JRDB015	*P. machaon kahli*	Canada: Manitoba: near Winnipeg; McCorkle, D.; 2005; 50.0014 -96.9167	KJ363272 hyb7		*	*
FS034	*P. zelicaon* x *machaon*	Canada: Alberta: Bragg Ck.: Fish Butte; Sperling, F.A.H.; 1987; on *Zizia aptera*; 50.91694 -114.53558	KJ363212 hyb9(M12)		*	*
FS151	*P. zelicaon x machaon*	Canada: Alberta: Buck Mtn.; Sperling, F.A.H.; 1987; on *Heracleum lanatum*; 53.05209 -114.73961	KJ363197 zel8(Z3)	KJ363303 zel8	*	*
FS201	*P. zelicaon x machaon*	Canada: Alberta: Buck Mtn.; Kondla, N.G.; 1990; 53.05209 -114.73961	KJ363228 zel9(Z3)		*	*
JRD103	*P. zelicaon* x *machaon*	Canada: Alberta: Buck Mtn.; Sperling, F.A.H.; 2010; 53.05209 -114.73961	KJ363277 zel7		*	*
JRD195	*P. zelicaon* x *machaon*	Canada: Alberta: Nordegg: Shunda Lkt.; Dupuis, J.R., Brunet, B.; 2010; 52.48259 -115.73825	KP262870 zel7		*	*
JRD197	*P. zelicaon* x *machaon*	Canada: Alberta: Nordegg: Shunda Lkt.; Dupuis, J.R., Brunet, B.; 2010; 52.48259 -115.73825	KP262871 zel10		*	*
JRD198	*P. zelicaon* x *machaon*	Canada: Alberta: Nordegg: Shunda Mtn.; Dupuis, J.R., Brunet, B.; 2010; 52.53151 -116.12610	KJ363278 zel10		*	*

JRD202	*P. zelicaon* x *machaon*	Canada: Alberta: Nordegg: Shunda Mtn.; Dupuis, J.R., Brunet, B.; 2010; 52.53151 -116.12610	KJ363279 zel10		*	*
JRD321	*P. zelicaon* x *machaon*	Canada: Alberta: Buck Mtn.; Dupuis, J.R.; 2011; 53.05209 -114.73961	KJ363280 zel7		*	*
JRD322	*P. zelicaon* x *machaon*	Canada: Alberta: Buck Mtn.; Dupuis, J.R.; 2011; 53.05209 -114.73961	KJ363281 zel10		*	*
JRD389	*P. zelicaon* x *machaon*	Canada: Alberta: Bragg Ck.: Fish Butte; Sperling, F.A.H., Sperling, E., Sperling, T.; 2011; 50.91694 -114.53558	KJ363283 hyb8		*	*
JRD391	*P. zelicaon* x *machaon*	Canada: Alberta: Bragg Ck.: Fish Butte; Sperling, F.A.H., Sperling, E., Sperling, T.; 2011; 50.91694 -114.53558	KJ363284 hyb8		*	*
JRD392	*P. zelicaon* x *machaon*	Canada: Alberta: Bragg Ck.: Fish Butte; Sperling, F.A.H., Sperling, E., Sperling, T.; 2011; 50.91694 -114.53558	KP262872 zel11		*	*
JRD393	*P. zelicaon* x *machaon*	Canada: Alberta: Bragg Ck.: Fish Butte; Sperling, F.A.H., Sperling, E., Sperling, T.; 2011; 50.91694 -114.53558	KJ363285 mach18		*	
JRD395	*P. zelicaon* x *machaon*	Canada: Alberta: Bragg Ck. Ski Hill; Dupuis, J.R.; 2011; 50.98175 -114.58286	KJ363286 zel10		*	*
JRD396	*P. zelicaon* x *machaon*	Canada: Alberta: Bragg Ck. Ski Hill; Dupuis, J.R.; 2011; 50.98175 -114.58286	KJ363287 mach18		*	*
JRD401	*P. zelicaon* x *machaon*	Canada: Alberta: Bragg Ck.: Mesa Butte; Dupuis, J.R.; 2011; 50.78071 -114.56134	KJ363288 mach18		*	
JRD640	*P. zelicaon* x *machaon*	Canada: Alberta: Kananaskis Country: Powderface Mtn.; Dupuis, J.R.; 2012; 50.84316 -114.84863	KJ363289 zel10		*	*
JRD641	*P. zelicaon* x *machaon*	Canada: Alberta: Kananaskis Country: Powderface Mtn.; Dupuis, J.R.; 2012; 50.84316 -114.84863	KJ363290 hyb8		*	*
JRD645	*P. zelicaon* x *machaon*	Canada: Alberta: Kananaskis Country: Powderface Mtn.; Dupuis, J.R.; 2012; 50.84316 -114.84863	KJ363291 mach18		*	
JRD681	*P. zelicaon* x *machaon*	Canada: Alberta: Bragg Ck.: Fish Butte; Sperling, F.A.H.; 2001; 50.91694 -114.53558	KJ363292 hyb10		*	*
JRD686	*P. zelicaon* x *machaon*	Canada: Alberta: Bragg Ck.: Fish Butte; Sperling, F.A.H.; 2001; 50.91694 -114.53558	KJ363293 hyb12		*	*

JRD386	*P. zelicaon* x *machaon (nitra)*	Canada: Alberta: Bragg Ck.: Fish Butte; Sperling, F.A.H., Sperling, E., Sperling, T.; 2011; 50.91694 -114.53558	KJ363282 mach18		*	*
FS143	*P. hospiton*	Sardinia; Crnjar, R. via Clarke, C.A.; 1989	*AF044009.1* hosp(H)	*AF044830.1* hosp		*
FS066	*P. indra indra*	USA: Washington: Wawawai co.: Wehling/Thompson culture; Wehling, W.; 1987	*AF044011.1* indra2(I)	*AF044824.1* indra1_1	*	*
FS181	*P. indra indra*	USA: Colorado: Jefferson co.: Lookout Mtn.; Fisher, M.S.; 1990; 39.7336 -105.2380	KJ363198 indra1(I)	KJ363304 indra1_3	*	*
FS267	*P. indra indra*	USA: Washington: Kittitas co.: N Fork Teanaway Riv; Peterson, M.A.; 1990; 47.2556 -120.8807	KJ363208 indra1(I)	KJ363314 indra1_2	*	*
FS197	*P. indra kaibabensis*	USA: Arizona: Coconino co.:13.2 mi W of Cameron; Griffin, B. via Emmel, J.F.; 1990; on *Lomatium parryi*; 35.8765 -111.6339	KJ363202 indra1(I)	KJ363308 indra2	*	*
FS238	*P. xuthus*	Japan: Tokyo Prefecture: Mount Takao; Taguchi, M. & Sperling, F.A.H.; 1990; 35.62496 139.24378	*AF043999.1* xuthus(X)	*AF044838.2* xuthus		

Table B. **Microsatellite loci used in this study** (from Zakharov & Hellman 2007). A "T" following the primer name indicates loci that were PIG-tailed (see methods). Abbreviations: T_a: annealing temperature, N: number of alleles observed, Range: allelic size range observed.

Locus	T_a (°C)	N	Range
PZ-A229T	57	15	203-239
PZ-B209T	56	11	188-208
PZ-B225T	57	23	210-300
PZ-B102T	57	24	173-247
PZ-A121	57	24	259-327
PZ-B12T	57	24	245-327
PZ-A110	57	17	217-255
PZ-A117T	57	32	249-357
PZ-A214	56	27	191-301
PZ-D224*	57	28	171-288

*primer sequences for PZ-D224 were misreported in Zakharov & Hellman (2007). Correct primer sequences are F(5'-3'): CACCATCATCAACAACAACC and R: TTGGTAGTGTTCCTTGACCAC.

Table C. **Primers for new sequences used in this study.** * corresponds to antisense or minority direction primers. Alternate names for primers are given in parenthesis. Reference positions are relative

to *Drosophila yakuba* (Clary & Wolstenholme 1985) for COI, and *Heliothodes diminutivus* (Cho *et al.* 1995) for EF-1α.

Gene	Name	Reference Position (3' end)	Sequence (5' → 3')
COI	Jerry	2183	CAA CAT TTA TTT TGA TTT TTT GG
	Mila*	2659	GCT AAT CCA GTG AAT AAT GG
	k741*	2578	TGG AAA TGT GCA ACT ACA TAA TA
	Pat*	3014	TCC AAT GCA CTA ATC TGC CAT ATT A
EF-1α	Bo (M44-1)	225	GCT CG(CT) GA(AG) CGT GGT ATC AC
	Juke (E600rc)*	620	CTC CTT ACG CTC AAC ATT CC
	Verdi3*	795	GAC ACC AGT TTC AAC TCT GCC

References

Cho S, Mitchell A, Regier JC, Mitter C, Poole RW, Friedlander TP, et al. A highly conserved nuclear gene for low-level phylogenetics: *elongation factor-1α* recovers morphology-based tree for heliothine moths. Mol Biol Evol 1995; 12(650-656).

Clary DO, Wolstenholme DR. The mitochondrial DNA molecular of *Drosophila yakuba*: Nucleotide sequence, gene organization and genetic code. J Mol Evol 1985; 22(252-271).

Sperling FAH, Harrison RG. Mitochondrial DNA variation within and between species of the *Papilio machaon* group of swallowtail butterflies. Evolution 1994; 48(408-422).

Zakharov EV, Lobo NF, Hellmann JJ. Introgression as a likely cause of mtDNA paraphyly in two allopatric skippers (Lepidoptera: Hesperiidae). Heredity 2009; 102(590-599).

Table D. (Next Page, after references) **Summary of morphological and ecological information pertinent to the species included in the present study.** Information was abstracted from the sources below:

Bird CD, Hilchie GJ, Kondla NG, Pike EM, Sperling FAH. Alberta butterflies. Edmonton: Provincial Museum of Alberta; 1995.

Chermock FH, Chermock RL. Two new races of Papilios from Manitoba. Bulletin of the Southern California Academy of Sciences. 1937;36: 11-13.

Ferguson, DC. The Lepidoptera of Nova Scotia. Part I: Macrolepidoptera. Nova Scotia: Nova Scotia Museum of Science Bulletin no. I; 1955.

Heitzman JR. A new species of *Papilio* from the eastern United States (Papilionidae). J Res Lepid. 1973;12: 1-10.

Klassen P, Westwood AR, Preston WB, McKillpp WB. The butterflies of Manitoba: Manitoba Museum of Man and Nature; 1989.

Layberry RA, Hall PW, Lafontaine JD. The butterflies of Canada. Toronto: University of Toronto Press; 1998.

Morris RF. Butterflies and moths of Newfoundland and Labrador: the macrolepidoptera. Quebec: Canadian Government Publishing Centre; 1980.

Murphy SM. Notes on *Papilio machaon aliaska* (Papilionidae) populations near Fairbanks, AK. J Lep Soc. 2008;62: 80-83.

Pelham J. Catalogue of the butterflies of the United States and Canada. J Res Lepid. 2008;40: xiv-658.

Remington CL. A new sibling *Papilio* from the Rocky Mountains, with genetic and biological notes (Insecta, Lepidoptera). Postilla. 1968;119: 1-40.

Schweitzer DF, Minno MC, Wagner DL. Rare, declining, and poorly known butterflies and moths (Lepidoptera) of forests and woodlands in the eastern United States. Washington DC: U.S. Forest Service, Forest Health Technology Enterprise Team; 2011.

Scott JA. The butterflies of North America: a natural history and field guide. Standford: Stanford University Press; 1986.

Sperling FAH. Evolution of the *Papilio machaon* species group in western Canada (Lepidoptera: Papilionidae). Quaestiones Entomologica. 1987;23: 198-315.

Tyler HA, Brown KS, Wilson KH. Swallowtail butterflies of the Americas: a study in biological dynamics, ecological diversity, biosystematics, and conservation. Gainsville, FL: Scientific Publishers Inc.; 1994.

Table D

Species	Subspecies	Distinctive wing characters	Flight period	Habitat	Host
P. machaon	Many subspecies across Eurasia, northern and western North America	Generally yellow background, pointed wing tips, long tails, eyespot connected to wing margin	Variable and habitat dependent	Generally strict hilltoppers	Asteraceae in arid or northern habitats, Apiaceae in mesic habitats
	aliaska	Flatter eyespot pupil, shorter tails and more rounded wing tips than other North American *P. machaon*	June-July	Alpine tundra and mountain tops	*Artemesia arctica, Cnidium cnidiifolium, Petasites frigidus*
	pikei	Similar to *P. m. oregonius*	June-July	Dry grassland and eroding banks	*Artemesia dracunculus*
	hudsonianus	Similar to *P. m. aliaska*, more club-shaped eyespot	June-July	Hilltops and boreal forest openings	*Heracleum lanatum* (?)*, Ligusticum scothicum, Petasites palmatus/frigidus, Zizia aptera* (?)
	dodi/bairdii/brucei/oregonius	Mostly yellow morph, but black morphs common in some regions	Multiple flights, variable; Alberta: May-August	Dry grassland and tops of high, eroding river banks	*Artemesia dracunculus*
P. polyxenes	Two subspecies in North America, others extending into South America	Generally black background (more yellow in American Southwest and South America), pointed wing tips, long tails, eyespot central, not connected to wing margin	Range dependent and variable	Variable, many hilltop, but also frequent open garden habitats in eastern North America	Wide variety of Apiaceae and occasional Rutaceae, often locally restricted

Table D (continued)

Species	Subspecies	Distinctive wing characters	Flight period	Habitat	Host
(P. polyxenes)	*asterius*	As above	Manitoba: late May-late June; North-east: late May-mid June and mid July to late September	As above	*Anethum graveolens, Angelica spp., Apium graveolens, Berula erecta, Carum carvi, Cicuta spp., Conium maculatum, Cryptotaenia canadensis, Cymopterus panamintensis, Daucus spp., Dictamnus albus, Foeniculum vulgare, Harboria trachypleura, Heracleum spp., Levisticum officinale, Ligusticum scothicum, Osmorhiza longistylis, Oxypolis canbyi, Pastinaca sativa, Petroselinum crispum, Ptilimnium capillaceum, Ruta graveolens, Sium suave, Spermolepis divaricata, Taenidia integerrima, Tauschia spp., Thamnosma spp., Thaspium barbinode, Zizia spp.*
	coloro	Often more yellow scaling ranging to yellow morph individuals	March-September (often rain dependent)	As above	*Cymopterus panamintensis, Daucus carota, Foeniculum vulgare, Lomatium parryi, Petroselinum crispum, Ruta graveolens, Tauschia spp., Thamnosma montana*
	americus/stabilis	Yellow background common	Many flights, close to all year	As above	*Apium leptophyllum, Spananthe paniculata, Cyclospermum leptophyllum*
P. zelicaon	Subspecies not currently recognized, geographic variation exists	Generally yellow background (regionally black), rounder wing tips, eyespot central, not connected to wing margin	Alberta: May-late July; south: March-September	Generally strict hilltoppers	*Anethum graveolens, Angelica spp., Apium graveolens, Carum carvi, Cicuta caulata, Citrus spp., Conioselinum scopulorum, Conium maculatum, Cymopterus spp., Daucus spp., Foeniculum vulgare, Harbouria trachypleura, Heracleum spp.* (incl. *lanatum*), *Ligusticum spp., Lomatium spp., Musineon tenuifolium, Oenanthe sarmentosa, Pastinaca sativa, Perideridia spp., Petroselinum crispum, Pimpinella spp., Pseudocymopterus montanus, Pteryxia spp., Ruta spp., Sium suave, Sphenosciadium capitellatum, Tauschia spp., Zizia aptera*

Table D (continued)

Species	Subspecies	Distinctive wing characters	Flight period	Habitat	Host
P. brevicauda	Three subspecies currently recognized, ecological characteristics do not differ between subspecies	Black background, rounded wing tips, shorter tails than most *P. machaon*	Mid June-late July	Hilltopping behavior common, particularly on ocean bluffs; also frequent lowland marsh and open garden habitats	*Angelica atropurpurea, Apium graveolens, Coelopleurum lucidum, Conioselinum chinense, Daucus carota, Heracleum lanatum, Ligusticum scothicum, Pastinaca sativa, Petroselinum crispum*
P. joanae	No subspecies recognized	Often indistinguishable from *P. polyxenes*, sometimes darker, with eyespot variably connected to wing margin	Multiple flights, May-September	Strictly flies under forest cover	*Taenidia integerrima, Thaspium barbinode, Zizia aurea*
P. m. kahli	*kahli* currently recognized as subspecies of *P. machaon*	Generally black background, pointed wing tips, long tails, eyespot connected to wing margin	Late May-late June	Variable, as in *P. polyxenes*	*Heracleum spp., Pastinaca sativa, Petroselinum crispum, Zizia spp.*
P. zelicaon x *machaon*		Generally yellow background (although black morphs relatively common), shorter, rounded wing tips, slightly shorter tails, eyespot extremely variable	May-July	Hilltopping behavior as in *P. zelicaon*	*Heracleum lanatum, Lomatium spp., Zizia aptera*
